# Autonomous Biogenesis of Early and Late Assembly Intermediates of a Large Ribosomal Subunit in a Minimal System

**DOI:** 10.1021/jacs.6c13587

**Published:** 2026-10-15

**Authors:** Noa Avidan, Michael Levy, Yuval Oren, Yoshihiro Shimizu, Roy H. Bar-Ziv, Shirley S. Daube

**Affiliations:** † Department of Chemical and Biological Physics, 34976 The Weizmann Institute of Science, Rehovot 7610001, Israel; ‡ Faculty of Biotechnology and Food Engineering, 26747 Technion—Israel Institute of Technology, Haifa 3200003, Israel; § Laboratory for Cell-Free Protein Synthesis, RIKEN Center for Biosystems Dynamics Research, Kobe, Hyogo 650-0047, Japan

## Abstract

Cell-free autonomous biogenesis of ribosomes is important for the bottom-up assembly of an artificial cell and for engineering of functional ribosomes with novel activities. The challenge lies in establishing conditions for a single-step multicomponent synthesis and assembly process in a minimal transcription–translation reaction. We previously reported autonomous synthesis and assembly of a bacterial small ribosomal subunit, but the assembly pathway of the large subunit is more complex with dozens of proteins that bind, fold, and chemically modify its ∼3000-base-long rRNA (rRNA). Here, we cosynthesized all the large subunit components including 33 ribosomal proteins (r-proteins) and 2 rRNAs from DNA brushes and followed the dynamics of assembly coupled to synthesis in real time using total internal reflection fluorescence microscopy. A systematic screen of the binding of each of the ribosomal proteins to the 23S rRNA revealed an assembly pattern indicative of early intermediates. The addition of genes coding for six rRNA assembly factors facilitated binding of late assembly ribosomal proteins, supporting formation of late-stage intermediates even in the absence of rRNA modifications. We established a scenario for the large subunit biogenesis via macromolecular colocalization that may aid in the identification and characterization of additional genes required for achieving complete functional ribosome self-regeneration in a minimal system.

## Graphical abstract

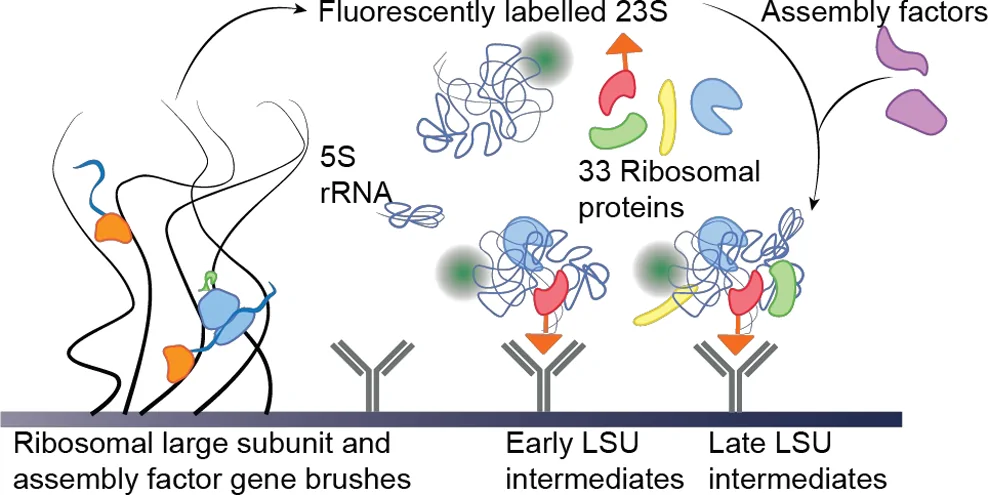

## Introduction

Ribosomes are the universal molecular machines that synthesize all proteins in all living cells. In prokaryotic cells, they are built in a fast and hierarchal self-assembly process coupled to synthesis and folding of three ribosomal rRNAs and dozens of r-proteins that constitute the small and large ribosomal subunits (SSU and LSU, respectively).^1,2^ Reconstituting ribosome biogenesis outside the cell in a minimal system would allow deciphering basic assembly mechanisms, engineering of ribosomes with diverse functions and properties, and establishing an artificial protein-based self-replicating system.^3–5^

Autonomous synthesis and assembly of all 35 components of the LSU in a minimal transcription–translation reaction would require expression of all the subunit genes as well as necessary assembly factors. In this work, we used the PURE system,^6^ which consists of a core of recombinant proteins: T7 RNA polymerase, the *Escherichia coli* ribosome, ten translation factors, and 20 synthetases, as well as tRNAs, amino acids, and nucleotides. In a bulk solution PURE reaction, with dozens of proteins expressed simultaneously, the concentration buildup takes about an hour, reaching a small fraction of micromolar per nascent protein. These concentrations are too low to drive assembly, compared to standard reconstitution assays of ribosomal subunits, commonly done with purified r-proteins and rRNA at the micromolar range.^7^

To circumvent the concentration barrier, we used a surface-bound reaction setup to locally concentrate all the nascent components, which we previously demonstrated with the biogenesis of the *E. coli* SSU in the PURE system.^8^ Twenty-seven different genes coding for the r-proteins, rRNA, and assembly factors of the SSU were immobilized on a glass surface as dense DNA brushes, forming boundary-free transcription–translation reaction centers that accumulate and colocalize the nascent ribosomal components for enhanced macromolecular interactions.^9^ The nascent SSUs were captured on the surrounding surface, spatially resolved from the ribosomes that synthesized them, and detected with high sensitivity by total internal reflection fluorescence (TIRF) microscopy, measuring the fluorescent signal of a broccoli aptamer^10^ encoded in the 16S rRNA. The assembly dynamics obtained using this methodology matched the established hierarchy and timeline of *in vitro* SSU assembly from purified components.^11,12^ Recently, we used the same approach to demonstrate simultaneous self-regeneration of all 30 translation proteins that are essential and sufficient for ribosome function in a minimal system. Each translation protein could be self-regenerated individually from its coding genes in a bulk solution reaction, but the simultaneous self-regeneration of all 30 proteins in one PURE reaction could only be achieved by colocalization of all the genes and the components of the reaction.^13^

There are additional challenges specific to LSU assembly. For decades, *in vitro* reconstitution of the *E. coli* LSU from purified native components has been performed as a two-step nonautonomous process in which both the temperature and the Mg^2+^ concentration were externally modified at nonphysiological conditions during the reaction,^14^ making it necessary to find conditions compatible with single-step autonomous biogenesis. It has recently been shown that the addition of two purified assembly factors, EngA and ObgE, suffices to achieve one-pot assembly of functional ribosomes from purified ribosomal components.^15^ Since the rRNA used in that study was purified from cells and included its native modifications, some of which have been shown to be essential for subunit assembly,^16–19^ more factors are likely required for LSU assembly using *in vitro* synthesized rRNA. Recently assembly of functional ribosomes from *in situ* synthesized rRNA, with either purified or nascent r-proteins, was demonstrated in an optimized *E. coli* cell-free extract for the integrated synthesis, assembly, and translation (iSAT) system,^20,21^ suggesting that the necessary factors are present in these cell-free extracts. Which genes are necessary and sufficient for autonomous LSU assembly and functionality when synthesized in a minimal system remains an open question.

Both the SSU and the LSU share a common assembly mechanism in which the 16S and 23S rRNA, respectively, serve as scaffolds for the sequential binding of the corresponding r-proteins, which help shape the rRNAs’ folding pathways. Therefore, we adapted the methodology we previously developed for cell-free SSU biogenesis to promote and monitor interactions between the 23S rRNA and the LSU r-proteins.^8^ We expressed all 33 r-proteins and 2 rRNAs from their respective genes immobilized as dense DNA brushes while monitoring the binding of *in situ* fluorescently labeled 23S (FL-23S) rRNA to surface-bound r-proteins in real time (Figure 1A). The gene composition in a single brush cluster dictated which r-protein bound to the surface in each reaction. Accumulation of the signal on the surface was monitored using TIRF microscopy (Figure 1B). We first scanned all the binary r-protein/rRNA interactions and found that most of the r-proteins classified as primary binders in the classical Nierhaus equilibrium map^22,23^ indeed exhibited early measurable signals. We then adjusted the biochemical conditions of the reaction to support multicomponent assembly with a side-by-side comparison of the interactions of six representative r-proteins with the rRNA either in a binary mode or with genes coding for all other LSU r-proteins. Using these optimized reaction conditions, we scanned all r-proteins/rRNA interactions in the multicomponent mode and found that most of the strong signals appeared earlier than the weak ones and corresponded to 23S structural domains (Figure 1C) that were identified as assembling in early stages by recent kinetic and structural studies.^24–26^ Lastly, in the presence of genes coding for the assembly factors EngA, ObgE, DbpA, DeaD, SrmB, and YhbY, r-proteins from structural domains known to assemble late interacted with the rRNA, suggesting that these factors are involved in the transition to late-stage intermediates.

**Figure 1:**
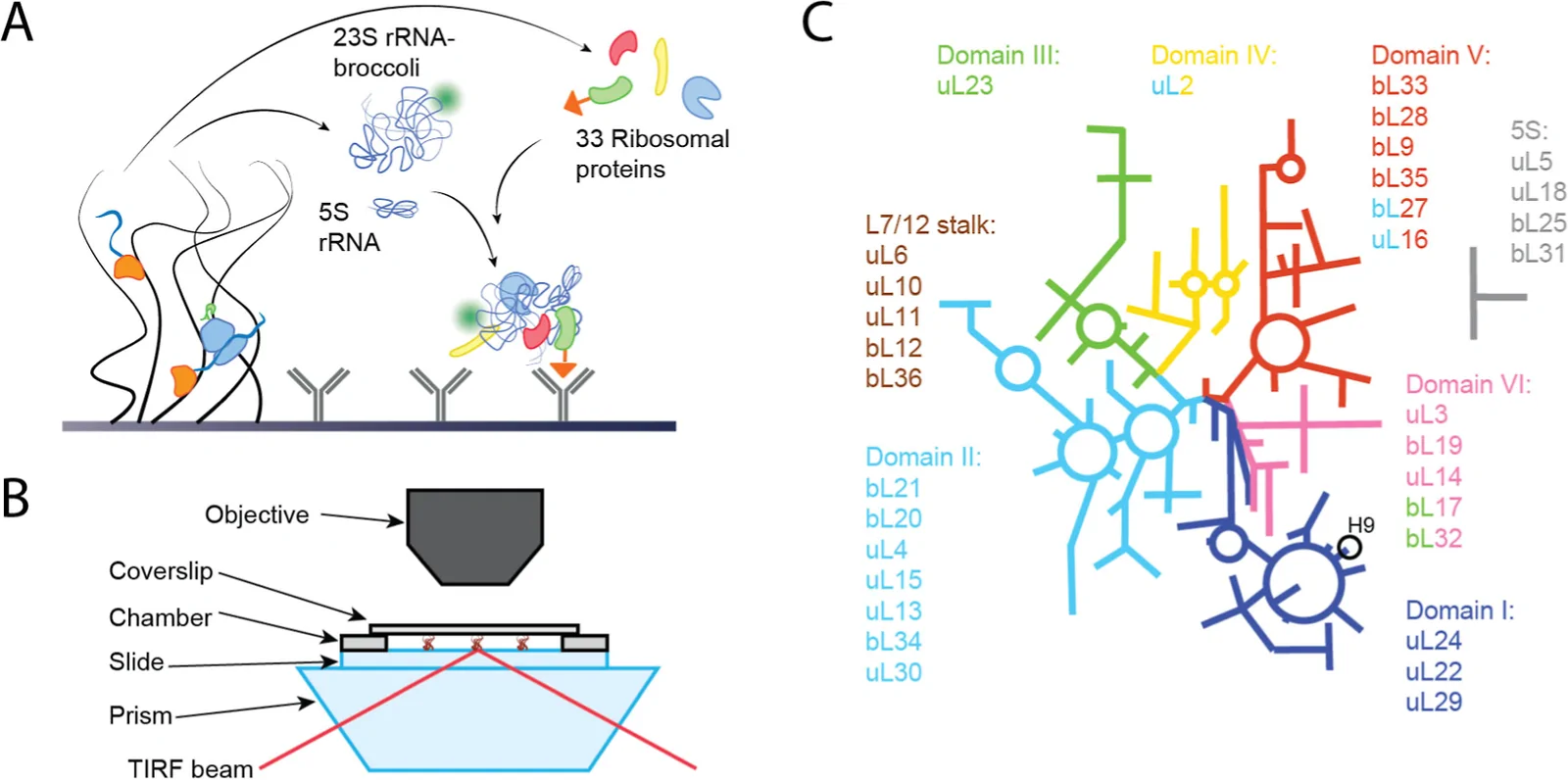
System setup for coupled synthesis and measurement of LSU r-protein interactions with the 23S rRNA. (A) Scheme of coupled synthesis and assembly of all LSU components in the PURE system. RNA polymerase (orange shape) and ribosomes (blue shape) express genes from the DNA brush immobilized on the surface of the reaction chamber. All nascent components are coexpressed locally, with one r-protein tagged with a hemagglutinin tag (orange triangle) and the 23S rRNA fluorescently labeled (green dot). Assembled intermediates are captured on the surface via surface-bound anti-HA antibodies (Y shapes), with surface fluorescence at the location of the antibodies indicating binding of the r-protein-HA to the 23S rRNA. (B) Clusters of DNA brushes (red) are immobilized on a fused silica slide fixed on a prism. The fluorescence signals accumulating on the surface are measured using prism TIRF (Supporting Information). (C) The structural domains identified in the 23S secondary structure^27^ and the r-proteins associated with each, as presented in^24^: domain I—dark blue, domain II—light blue, domain III—green, domain IV—yellow, domain V—red, domain VI—pink, 5S—gray, and the L7/L12 stalk region—brown, with the L7/L12 stalk region considered separately from the rest of domain II for convenience. This color assignment was used in the remaining panels and figures. For simplicity, the colors of r-proteins that are structurally associated with two domains (uL2, uL16, bL17, bL27, and bL32) were depicted in the remaining panels and figures by the color of the domain they were first observed in the structural studies.^24^ The H9 helix of the 23S rRNA is marked by a circle. Scheme adapted from ref 24. Available under a CC-BY 4.0 license. Copyright 2023, Springer Nature.

## Results

### Monitoring Cosynthesis and Interactions of 23S rRNA with Each of the LSU r-Proteins

As an initial test for our cell-free LSU biogenesis assay, we attempted to characterize the coupled synthesis and binary interactions of the 23S rRNA with each of the LSU r-proteins in the absence of any other r-proteins or assembly factors (Figure 2A). Toward that, we inserted a hemagglutinin peptide tag (HA) at the C-terminus (C-ter) of each r-protein and confirmed that each of the 33 tagged r-protein-HAs could be synthesized fully from DNA brushes and bind to a surface covered with anti-HA antibodies (Supporting Information, Figure S1), demonstrating that the C-ter HA tag was accessible for surface binding.

**Figure 2:**
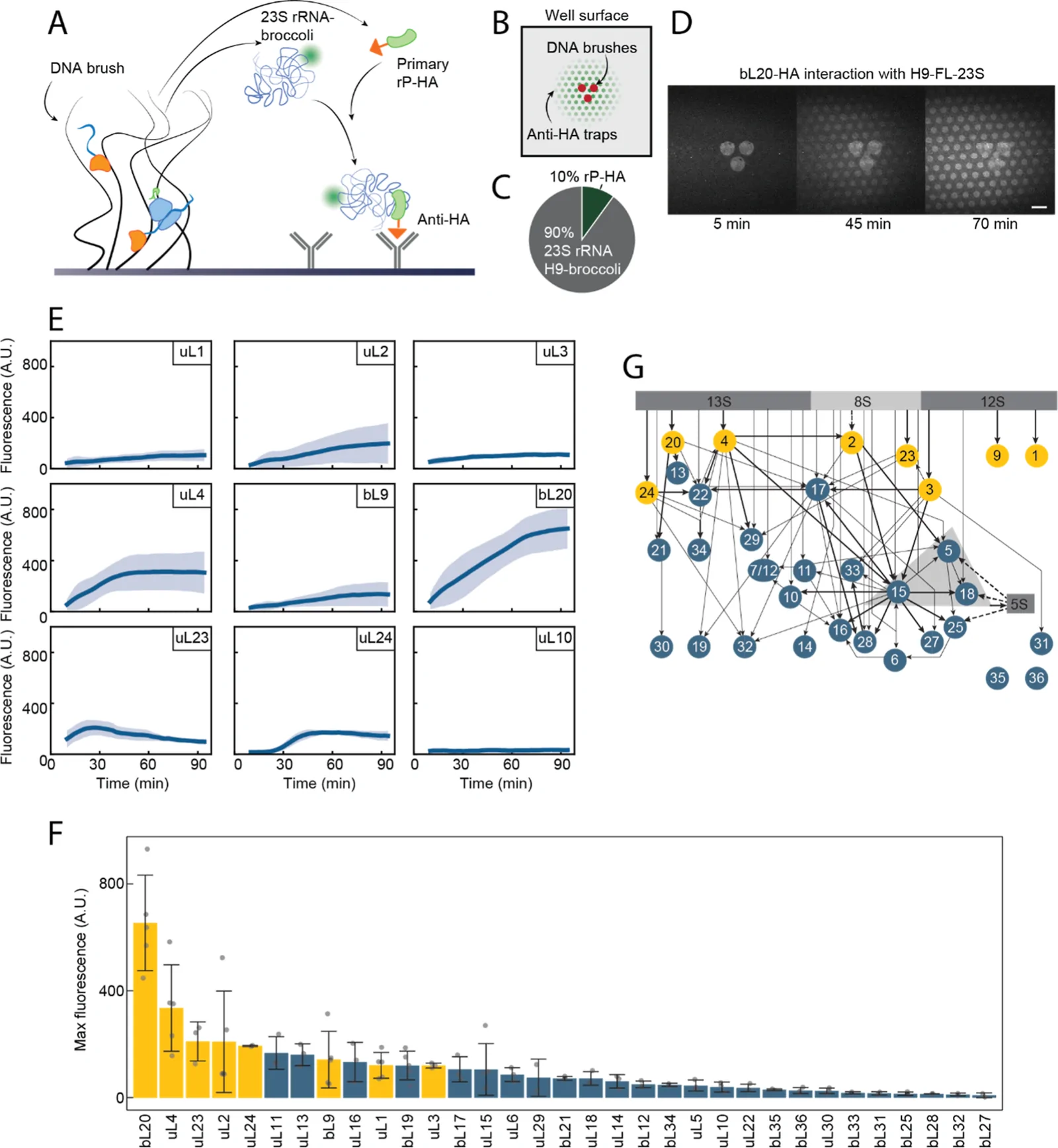
Coupled synthesis and binary interaction of each LSU r-protein with the 23S rRNA. (A) Scheme of synthesis and interaction of the H9-FL-23S construct with an HA-tagged r-protein and surface capture as in Figure 1A but without the r-proteins’ genes. (B) Scheme of three identical DNA brushes (red circles) coding for the H9-FL-23S rRNA and HA-tagged r-protein genes surrounded by the anti-HA antibodies patterned in a hexagonal grid (green array). (C) Composition of genes in each DNA brush; the H9-FL-23S and HA-tagged r-protein genes were encoded in the DNA brushes at a 9:1 ratio. (D) Three TIRF images with 488 nm excitation monitoring the interaction of primary binder bL20-HA with the H9-FL-23S rRNA after 5, 45, and 70 min from the start of the reaction (Supporting Information). The three DNA brushes are visible in the center of the image upon H9-FL-23S rRNA synthesis, and the signal spreading “lights up” the patterned antibodies with time. Scale bar: 100 μm. (E) Averaged dynamic plots of the H9-FL-23S rRNA TIRF signal surrounding 9 r-protein-HAs: uL1, uL2, uL3, uL4, bL9, bL20, uL23, and uL24, categorized as primary binders, and uL10 as a reference late binder.^22^ The nonaveraged dynamic plots of all the r-protein-HAs are in Figure S3. (F) The average max. Fl. signal and standard deviation across at least 3 repeats measured for each r-protein-HA binding to the FL-23S rRNA, based on the raw data in Figure S3. The primary binders are colored yellow for a simple comparison with the Nierhaus thermodynamic assembly map shown in (G), which depicts the binding dependencies of the *E. coli* 50S ribosomal proteins in LSU reconstitution experiments.^22,23^ Scheme adapted from ref 23. Available under a CC-BY 4.0 license. Copyright 1987, Elsevier.

For *in situ* fluorescence labeling, we chose the hairpin loop of the H9 helix of the 23S rRNA for insertion of the broccoli aptamer sequence into the rRNA coding gene due to its location in an external region of the ribosome, minimizing potential steric hindrance (Supporting Information, Figures 1C and S2). In addition, both the H9-FL-23S and 5S rRNA gene constructs included a 3′-HDV ribozyme sequence to ensure that most of the transcripts had a precise 3′ end^28^ (Supporting Information, Figure S2A,B). To finalize the initial characterization of the reaction setup, we prepared an additional control construct in which the broccoli aptamer was fused to the 3′ end of the 23S rRNA to verify qualitatively that a substantial fraction of the 23S rRNA, synthesized either in bulk solution reactions or from surface-bound genes, included the 3′ end (Supporting Information, Figure S2B,D–G).

We immobilized clusters of three identical DNA brushes on the glass surface of a reaction chamber, each cluster containing a gene coding for a different r-protein-HA mixed with the gene coding for the H9-FL-23S rRNA at a 1:9 ratio (Figure 2B,C). This ratio, found empirically for the SSU biogenesis assay, avoids surface saturation by the r-protein-HA, which might lead to sequestration of rRNA/r-protein-HA complexes into the bulk when there are no vacant surface binding sites.^8,9^ Upon addition of the PURE expression system to the chamber, a fluorescence signal appeared in the brushes, indicating synthesis of the H9-FL-23S (Figure 2D, 5 min). With time, a hexagonal fluorescence pattern emerged around some of the brush clusters, indicative of H9-FL-23S binding to the anti-HA antibodies within each hexagon, mediated by the r-protein-HA (Figure 2D, 45 min, 70 min). The hexagonal pattern was created by surface lithography prior to anti-HA antibody immobilization (Supporting Information), providing a local background throughout the surface for sensitive detection. In addition, by capturing the fluorescence signals only within ∼ 100 nm above the surface via TIRF microscopy, we achieved further background reduction (Figure 1B).

The signal spread away from the brush clusters with a lag time, a rise in signal, and saturation (Figures 2E and S3). We calculated the maximum fluorescence signal (max. Fl.) for each r-protein-HA cluster (Supporting Information, Figure 2F) and noticed a large variation among replicates, most likely due to the overall low signals and the convoluted rates of rRNA and r-protein synthesis, macromolecular association, diffusion, and surface binding. Therefore, we interpreted the data only qualitatively and noted a correspondence with the thermodynamic assembly map that had been established with purified LSU components, identifying some r-proteins as primary binders to the 23S rRNA, binding independently of other r-proteins, and some as late binders, contingent on prior binding of other r-proteins^14^ (Figure 2G). Most of the previously identified primary binders, such as uL2-HA, uL4-HA, bL20-HA, uL23-HA, and uL24-HA, had the highest signals in our measurements, and most of the late binders, such as bL10, bL35, and bL36, had barely detectable signals (Figures 2E,F and S3). This qualitative correlation was encouraging but suggested that the reaction parameters should be adjusted before attempting the simultaneous biogenesis of all LSU components.

### Simultaneous Synthesis of 33 LSU r-Proteins Coupled to 23S rRNA Binding

Anticipating even lower signals during synthesis of all LSU components simultaneously, we attempted to improve the reaction conditions by supplementing the PURE reaction with the crowding agent PEG 8000 (4%) to enhance macromolecular interactions^29,30^ and with purified protein chaperones GroE and DnaK to assist in proper folding of the nascent r-proteins (Supporting Information). We also increased the number of identical DNA brushes in each cluster from 3 to 20 for an enhanced signal. Each brush encoded the H9-FL-23S rRNA, one r-protein-HA, 32 nontagged r-proteins, and the 5S rRNA (Figure 3A–C). The effect of the r-proteins was tested by replacing their genes with noncoding DNA to maintain a similar DNA brush density (Supporting Information, Figure 3C). We further calibrated empirically, using the r-protein uL22-HA, the gene ratio between the FL rRNA, the r-protein-HA, and the nontagged r-proteins to find the smallest ratio between the 23S and the nontagged r-proteins that yielded an appreciable fluorescence signal on the surface (Figure S4). On the basis of this analysis, we used a gene ratio of 1:5:30:64 r-protein-HA/5S rRNA/23S rRNA/r-proteins in the following experiments, with the ratio of 5S and 23S rRNA genes proportional to their relative lengths (Figure 3C).

**Figure 3:**
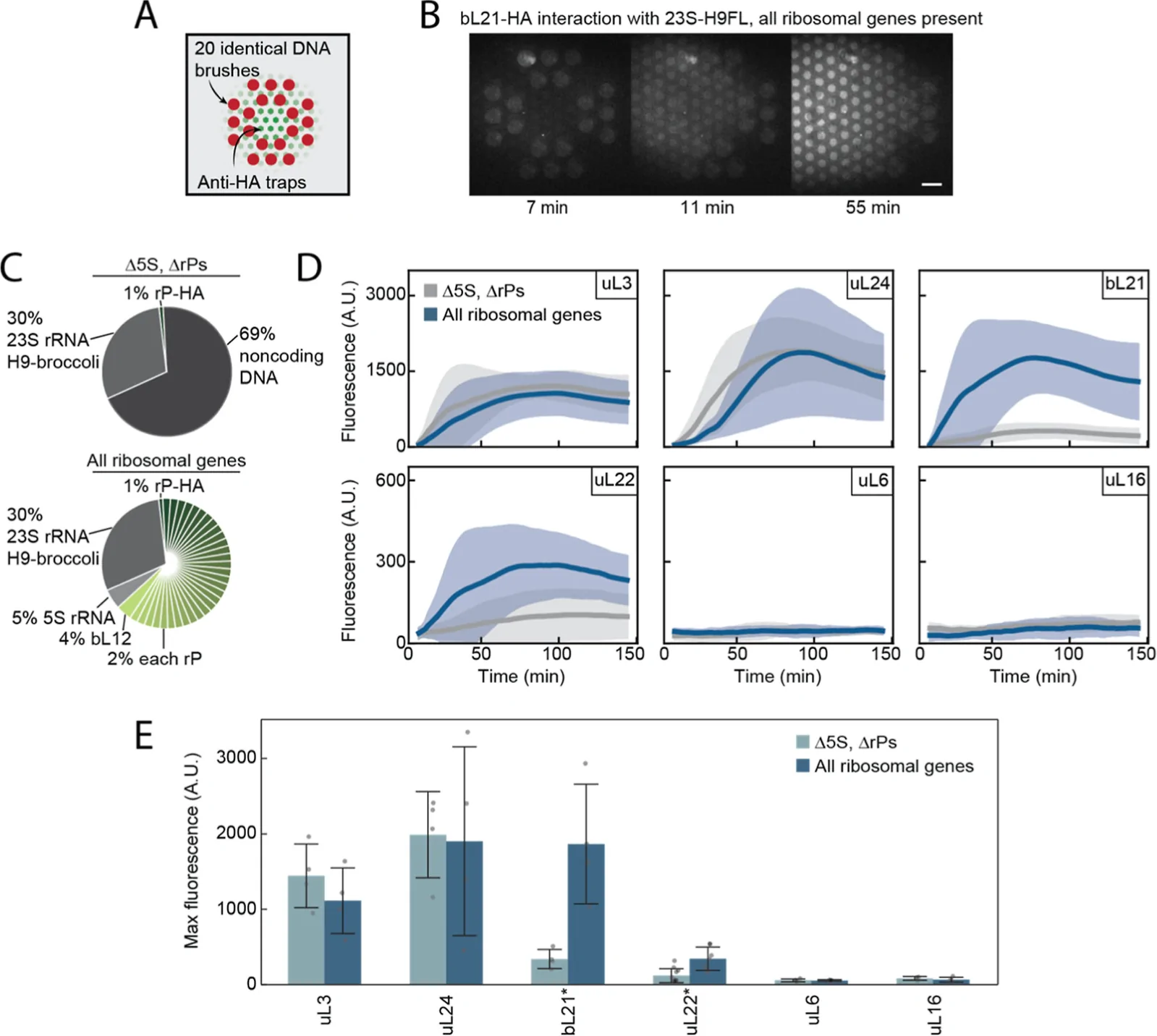
Multicomponent conditions for simultaneous coexpression of FL-H9-23S rRNA with all r-proteins. (A) Arrangement of 20 identical DNA brushes, surrounded by anti-HA antibodies as in Figure 2B. (B) Three TIRF images monitoring the interaction of bL21-HA with the H9-FL-23S rRNA in the presence of genes coding for all ribosomal components after 7, 11, and 55 min from the start of the reaction. The PURE mix was supplemented with 4% PEG 8000 and protein chaperons (Supporting Information). The 20 DNA brushes are visible with 488 nm excitation due to Fl-H9-23S rRNA synthesis within the brushes. With time, the fluorescence signal spreads and accumulates on the patterned antibodies. Scale bar: 100 μm. (C) Composition of genes in each DNA brush, testing coupled synthesis and assembly of all ribosomal components (bottom, 30% H9-FL-23S, 5% 5S rRNA, 1% HA-tagged r-protein, and 64% an equal mix of the other r-proteins) compared to the same brush composition with r-proteins and 5S genes replaced by noncoding DNA (top). In both cases, the DNA mixes were spotted on the well surface in a 20-brush configuration as shown in (B) and expressed under identical biochemical conditions. (D) Averaged dynamics of the H9-FL-23S TIRF signal and (E) the max. Fl. signal of the dynamics over 100 min for uL3-HA, uL24-HA, bL21-HA, uL22-HA, uL6-HA, and uL16-HA in the presence (dark blue) and absence (light blue) of the rest of the r-proteins. Standard deviations are across 4–8 repeats (dynamics of the replicas are shown in Figure S5). Asterisks by the r-protein names indicate a statistically significant difference between the two conditions for the given r-protein (Table S3).

We tested these optimized assembly conditions by conducting a side-by-side comparison of 23S-rRNA binding to six r-protein-HAs with and without all of the r-proteins. We monitored the signal accumulation on the surface with time, plotted the dynamics of at least 3 replicates (Figure S5), and calculated the average dynamics and the max. Fl. signal (Supporting Information, Figure 3D,E). Of the six r-proteins, uL3-HA and uL24-HA had high max. Fl. values both with and without the genes coding for the other r-proteins, in agreement with their clear role as primary binders in the classical assembly map (Figure 2G). For bL21-HA and uL22-HA, there was a statistically significant enhancement of the binding to the H9-FL-23S rRNA by the genes coding for all the r-proteins and no effect on the late r-proteins uL6 and uL16, which did not bind to the 23S under any condition. (Statistical analysis is detailed in Supporting Information and in Table S3.) Furthermore, the assembly supporting conditions of this experiment abolished completely the low binding of uL6-HA and uL16-HA to the 23S rRNA that was initially observed in the binary interaction mode (Figure 2F), suggesting that the inclusion of the protein chaperones and PEG was important to obtain specific binding. We further validated that this effect on uL6-HA and uL16-HA was not due to the influence of PEG on their expression efficiency in the PURE system (Figure S6).

We then applied these conditions to all of the r-protein-HA clusters aside from bL12-HA, which was excluded due to its tetrameric structure, possibly complicating signal comparison to the other monomeric LSU-r-proteins when bound to the surface.^31^ We measured the accumulation of the H9-FL-23S fluorescence signal on the surface for each r-protein-HA cluster, plotted the dynamic curves of at least 3 replicates for each (Figure S7), and displayed the calculated average max. Fl. signals (Supporting Information, Figure 4A) arranged in groups according to their association with the different structural domains of the 23S rRNA (Figure 1C). We observed that for the structural domains I, II, III, IV, and VI, more than half of the r-proteins associated with each domain had appreciable max. Fl. signals (uL22, uL24, and uL29, 3/3 of domain I; uL4, uL13, uL15, bL20, and bL21, 5/7 of domain II; uL23, 1/1 of domain III; uL2, 1/1 of domain IV; and uL3, bL17, and bL19, 3/5 of domain VI). For the rest of the structural domains, there was almost no detectable fluorescence signal for their respective r-protein-HA: the domain associated with the 5S rRNA (uL5, uL18, bL25, and bL31, 4/4), domain V (uL1, bL9, uL16, bL27, bL28, bL33, and bL35, 7/7), and the L7/12 stalk (uL6, uL10, uL11, and bL36, 4/4) (Figures 4A and S7).

**Figure 4:**
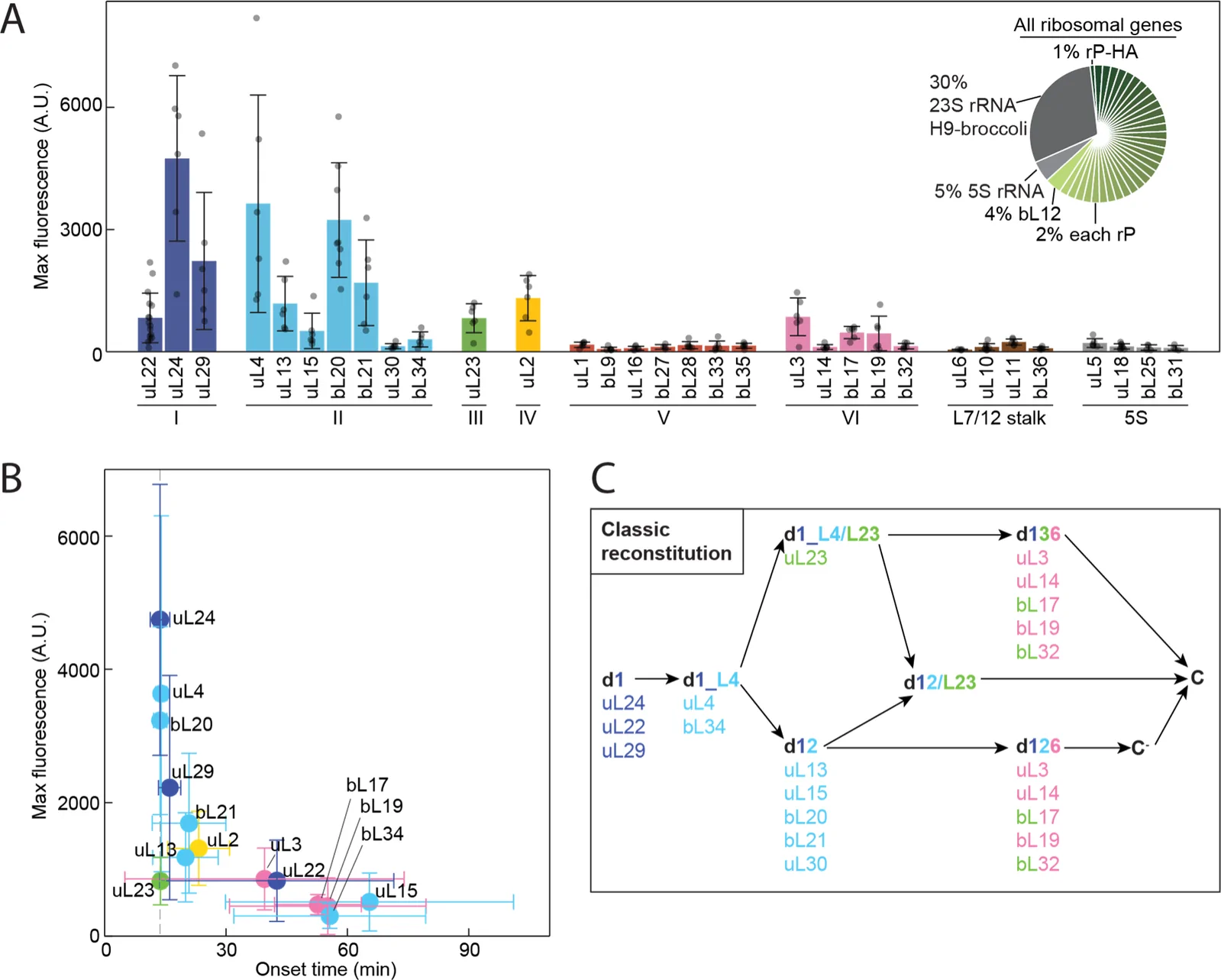
Coupled synthesis and assembly of all LSU components. (A) The average max. Fl. signal and standard deviation across at least 4 repeats measured for each r-protein-HA in the presence of all r-protein genes based on the raw data in Figure S7. Inset: the gene ratio in each DNA brush was 30% H9-FL-23S, 5% 5S rRNA, 1% HA-tagged r-protein, and 64% an equal mix of the other r-proteins. The PURE mix was supplemented with 4% PEG 8000 and protein chaperons (Supporting Information). (B) The max. Fl. signal as a function of onset time (Supporting Information) is presented per r-protein, for those r-proteins with a positive signal above the background threshold (the average onset times are shown in Figure S7). The error bars represent the standard deviation across the positive repeats. The dashed gray line represents the starting time of the measurement, with r-proteins with onset times plotted close to the starting point showing a fluorescent signal at the start of the measurement. (C) Scheme rendering published *in vitro* assembly timelines based on cryo-EM analysis of early assembly intermediates of the *E. coli* LSU during classical ribosome reconstitution from purified components.^24^ Scheme adapted from ref 24. Available under a CC-BY 4.0 license. Copyright 2023, Springer Nature.

We further measured the time interval between the reaction starting point (defined as the time point at which the temperature of the device reached 30 °C) and the time of first appearance of the fluorescence hexagonal pattern and defined it as the onset time (Supporting Information, Figure S8). For some r-protein-HAs (uL24 and uL29 from domain I; uL4 and bL20 from domain II; and uL23 from domain III), the hexagonal pattern was already observable at the start of the measurement, suggesting a rapid assembly. We plotted the max. Fl. signal as a function of the onset time for r-proteins associated with domains I, II, III, IV and VI (Figure 4B) and observed that all r-proteins associated with domain I and several r-proteins associated with domain II had higher signals and an earlier onset time than those associated with other domains, suggesting that these domains were assembled early in the cell-free biogenesis reaction. Several r-proteins associated with domains I (uL22), II (bL34 and uL15), and VI (uL3, bL17, and bL19) bound the 23S rRNA as a delayed group, with onset times longer than 30 min (Figure 4B).

The classification of the r-proteins with appreciable max. Fl. values and short onset times corresponded well with the recent cryo-EM characterization of early LSU intermediates assembled in the classical approach from purified ribosomal components (summarized in Figure 4C),^24^ suggesting that assembly of early intermediates had taken place, with minor deviations: uL30 had a very low max. Fl. value, suggesting involvement at a rather late stage. Our observed uL30 late assembly resembles its involvement in a recent characterization of early assembly intermediates produced cotranscriptionally in the iSAT reaction.^25^ bL34 had a low max. Fl. and a rather long onset time, which do not correspond to its participation in very early intermediates of either the classical or the iSAT reactions. uL2 had an appreciable max. Fl. value and short onset time, unlike its classification in late intermediates in both the classical and iSAT reactions, but in agreement with its classification as a primary binder in the Nierhaus assembly map (Figure 2G).

Overall, our results so far suggested that when the ribosomal components were expressed in the PURE system, LSU biogenesis did not progress past the assembly of the early intermediates. This was expected since the minimal system lacks any assembly factors or rRNA-modifying enzymes, and the coupling to gene expression reaction conditions dictated a constant Mg^2+^ concentration and temperature. The identification of early intermediates suggested that the addition of genes coding for assembly factors could facilitate progression to late intermediates.

### Genes Coding for Two GTPase Factors Facilitate Biogenesis of Late LSU Intermediates

It has recently been shown that the addition of two LSU assembly factors, ObgE and EngA, was sufficient to achieve one-step reconstitution of functional ribosomes from purified components in the PURE system.^15^ We hypothesized that LSU biogenesis could progress to late-assembly intermediates by including the genes coding for EngA and ObgE. We added the genes as separate DNA brushes, allowing us to explore a wide range of assembly factor gene concentrations without changing gene concentrations of the ribosomal components (Figure 5A,B). The 23S and 5S rRNA genes were replaced with a single DNA construct, containing genes coding for both rRNA molecules in a back-to-back configuration (Supporting Information, Figure S2A) to improve rRNA synthesis levels for late stages of assembly, as the 5S is part of a late-binding domain. Finally, we verified that ObgE and EngA were expressed in bulk PURE reactions (Figure S6) and found that EngA was expressed poorly compared with ObgE at all PEG concentrations tested (0, 2, and 4%).

**Figure 5:**
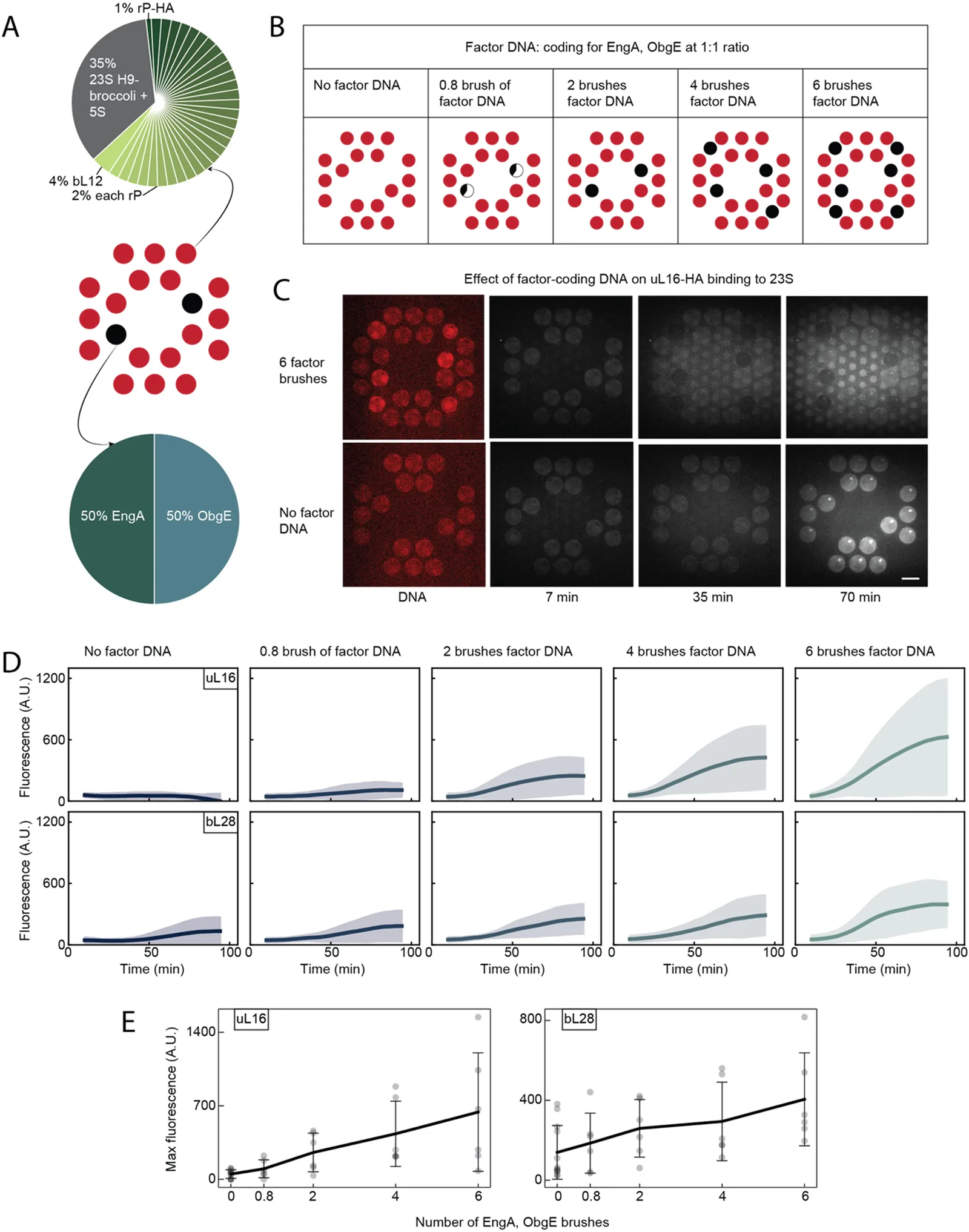
The effect of EngA and ObgE genes on the coupled synthesis and assembly of late r-proteins uL16 and bL28. (A) DNA mixes containing the genes coding for all LSU components (top) were spotted in an 18-brush configuration (center, red), along with brushes (center, black) coding for the factors EngA and ObgE at a 1:1 ratio (bottom). (B) Schemes of the DNA brush arrangements in each configuration: 18 identical brushes (in red) coded for the ribosomal components and a differing number of brushes (black) coded for the assembly factors. (C) Four TIRF images monitoring the interaction of uL16-HA with the H9-FL-23S rRNA after 7, 35, and 70 min after the start of the reaction, with (top row) and without (bottom row) six assembly factor brushes. The PURE mix was supplemented with 2% PEG 8000 and protein chaperons (Supporting Information). The image on the left is 647 nm emission depicting the ATTO-647-labeled DNA. Scale bar: 100 μm. (D) Averaged assembly kinetics along with the standard deviation at each time point for two r-proteins, uL16 (top) and bL28 (bottom), with a differing number of DNA brushes coding for the factors EngA and ObgE, ranging from 0 factor brushes to 6. Each panel contains 6–12 repeats for each condition, based on the raw data in Figure S9. (E) The average max. Fl. signal and standard deviation across all repeats of the dynamic plots in (D).

In the same reaction chamber, we immobilized brush clusters coding for all r-proteins and the H9-FL-23S and 5S rRNAs with either uL16-HA or bL28-HA. Both r-proteins are in domain V and had barely detectable fluorescence signals in the absence of assembly factors (Figure 4A). The addition of 0.8, 2, 4, and 6 DNA brushes composed of EngA and ObgE genes at a 1:1 ratio resulted in a statistically significant increase in the H9-FL-23S signal accumulated on the surface for both uL16-HA and bL28-HA, with higher amounts of the genes coding for the factors resulting in a higher binding signal (Figures 5C–E and S9), suggesting that these factors enabled progression to later assembly stages.

We tested the effect of EngA and ObgE on H9-FL-rRNA binding of more r-proteins, namely, uL6-HA, bL9-HA, uL22-HA, bL32-HA, and bL36-HA, by adding their genes in 4 DNA brushes and comparing them to the absence of their genes (Figure 6A,B). uL22 served as a reference r-protein that assembled in an early intermediate and was therefore expected to be unaffected by the assembly factors. Indeed, the rRNA binding of the late binders uL6-HA, bL9-HA, bL32-HA, and bL36-HA increased in the presence of the genes coding for the assembly factors, but the binding of uL22-HA to the 23S rRNA did not seem to be affected in a statistically significant manner (Figures 6C and S10), supporting the notion that EngA and ObgE participate in late stages of assembly. We further tested the effect of 4 additional RNA helicases and maturation factors (DbpA, DeaD, SrmB, and YhbY) that have been implicated in LSU assembly^1^ on the binding of the late binders uL14 and uL16, compared to the early binder bL21 as a reference. We first verified the expression of DbpA, DeaD, SrmB, and YhbY in bulk reactions (Figure S6) and then added their genes as separate brushes to the clusters of ribosomal component genes with an HA tag on uL14, uL16, and bL21. We observed stimulation of H9-FL-23S rRNA binding for uL14-HA and uL16-HA, with a magnitude similar to the effect of EngA and ObgE, and no statistically significant effect on the early r-protein bL21-HA (Figures 6D,E and S11). Altogether, these findings support an LSU assembly mechanism in which late assembly intermediates beyond the core of the LSU could be obtained in the autonomous biogenesis setup by simply adding genes encoding assembly factors required for their formation.

**Figure 6:**
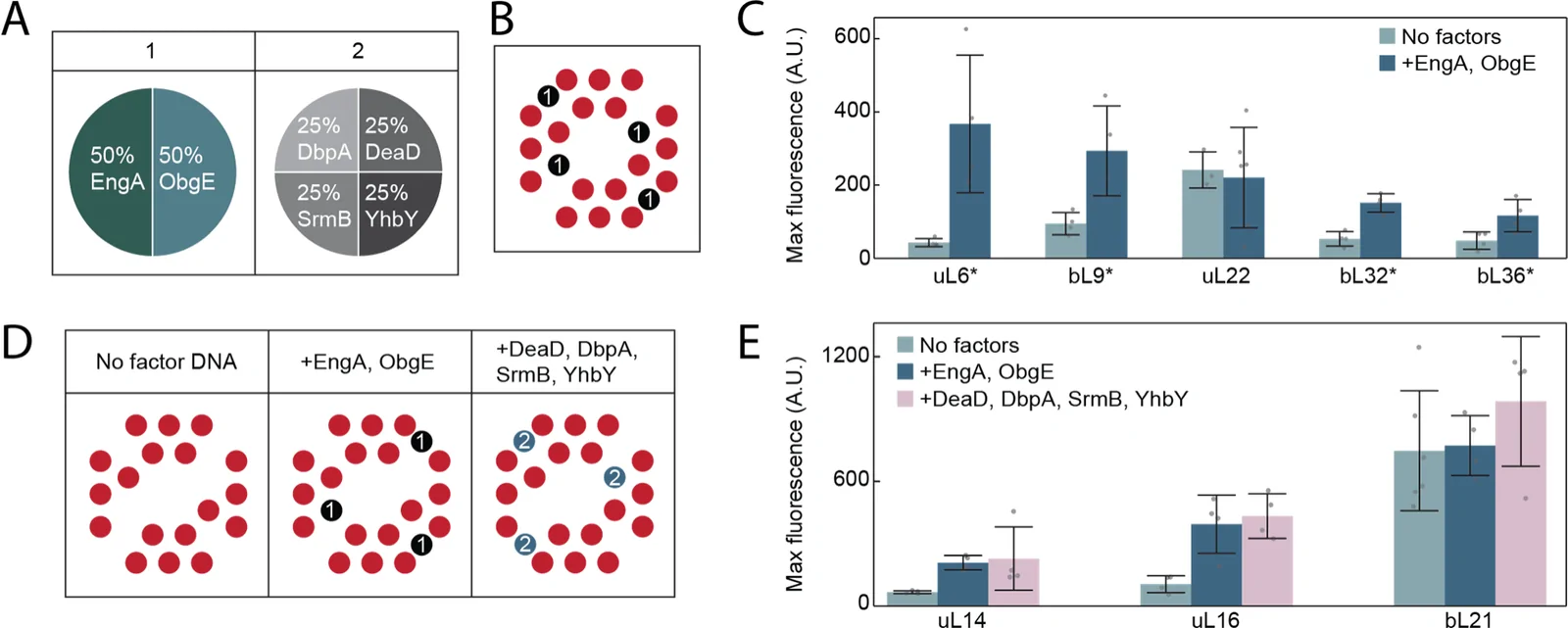
The effect of several assembly factors on the coupled synthesis and assembly of late r-proteins. (A) Two sets of assembly factors were used in these experiments: Set 1 of EngA and ObgE and Set 2 of DbpA, DeaD, SrmB, and YhbY at a 1:1 ratio. (B) Scheme of a DNA brush arrangement containing 18 identical brushes (red) coding for the LSU components and 4 brushes (black) coding for the assembly factors of Set 1. (C) Max. Fl. signal over 100 min and standard deviation across 4 repeats of the dynamics corresponding to the binding of uL6-HA, bL9-HA, uL22-HA, bL32-HA, and bL36-HA to the Fl-H9-23S rRNA with 0 (light blue) or 4 (dark blue) brushes coding for the Set 1 factors in (A), based on the raw data in Figure S10. The PURE mix was supplemented with 2% PEG 8000 and protein chaperons (Supporting Information). (D) Schemes of DNA brush arrangements containing 18 identical brushes (red) coding for the ribosomal components and 3 brushes coding for either the Set 1 or the Set 2 factors. (E) Max. Fl. signal over 100 min and standard deviation across 4 repeats of the dynamics corresponding to the binding of uL14-HA, uL16-HA, and bL21-HA to the Fl-H9-23S rRNA with no assembly factors (light blue), with Set 1 (dark blue) or with Set 2 (pink), based on the raw data in Figure S11. The PURE mix was supplemented with 2% PEG 8000 and protein chaperons (Supporting Information).

## Discussion and Conclusions

Altogether, we have laid the foundation for a platform aiming for coupled synthesis and assembly of the *E. coli* LSU in the minimal PURE system. We demonstrated the expression of all components of the subunit in the PURE system and showed that interactions of the r-proteins with the 23S rRNA take place during coupled synthesis from the same DNA brush. In the absence of assembly factor genes, the conditions were not sufficient for the full subunit assembly. However, most of the r-proteins associated with domains I, II, III, and VI were observed to bind to the 23S rRNA, implying that most of the core of the LSU was formed, in agreement with previous data on early LSU intermediates and r-protein binding order.^24^, Some differences were observed, for example, uL22 bound the r-RNA later than the other r-proteins associated with domain I, and uL4 and bL20 bound at short onset times similarly to the domain I r-proteins uL24 and uL29, in contrast with longer onset times for other domain II r-proteins such as bL34 and uL15 (Figure 4C). This may be a consequence of the coupling of assembly to synthesis, since in a biogenesis scenario, the concentration of the r-proteins starts from zero and accumulates over time. Thus, primary binders such as uL4 and bL20 may bind to the 23S as soon as they reach a high enough concentration to facilitate their assembly, while nonprimary binders such as uL22 and bL34 require sufficient synthesis of themselves as well as the other r-proteins required for their assembly.

We then showed that the addition of genes coding for EngA, ObgE, DbpA, DeaD, SrmB, and YhbY to the surface-based synthesis and assembly assay facilitated the assembly of several late binders to the 23S rRNA, driving progress toward later stages of ribosome biogenesis. By simply adding genes to the DNA brushes, this platform could be modified in the future to express additional assembly factors to aid in LSU folding and assembly and rRNA-modifying enzymes that would modify the 23S rRNA. Additionally, increasing 5S synthesis could enhance the assembly of late-binding r-proteins, particularly the 5S-associated r-proteins and domain V r-proteins, whose assembly may be improved by the integration of the 5S into the structure as well as the assembly factors.

The LSU biogenesis in surface-localized reactions displayed some interesting properties. First, while ribosome assembly in prokaryotic cells takes about 2–3 min,^32^ the time scales of LSU assembly in the surface-based system appeared to be in the range of 30 min to an hour, similar to what we observed for SSU synthesis.^8^ This may be related to the cumulative time for concentration buildup necessary for assembly and free diffusion over tens of micrometers to bind the surface. Although the data represent ensemble averages, we observed large variability as indicated by the error bars, which could stem from a number of reasons: (i) absence of compartmentalization leading to variations in the number of measurable bound intermediates near the sites of synthesis; (ii) a complex multicomponent kinetic process that is not synchronized; and (iii) small changes in parameters may result in major effects due to competing multiple pathways of assembly leading to a broad distribution of time scales and yield of intermediates, as the reaction resources are depleted. Such effects may be mitigated by optimizing the assay to obtain more robust synthesis and assembly of these intermediates in the PURE system by (i) compartmentalizing the reactions, (ii) creating delays in rRNA synthesis to allow sufficient accumulation of r-proteins, and (iii) adding more assembly factors to prevent kinetic traps.

## Supplementary Material



## Data Availability

The source data generated in this study and the analysis have been deposited in the Zenodo database under accession code 10.5281/zenodo.21820647

## Notes

Detailed experimental procedures including the description of DNA preparations, total internal reflection fluorescence (TIRF) microscopy and setup preparation, reconstituted *in vitro* reactions on a chip, silicon chip fabrication and protein expression in silicon compartments, gel electrophoresis analysis of *in vitro* synthesized rRNA and proteins, data analysis including plotting average dynamic curves, onset time calculation, statistical analysis, and additional experimental details (PDF)
