# Supplementary material for: Autonomous Biogenesis of Early and Late Assembly Intermediates of a Large Ribosomal Subunit in a Minimal System
Source: J Am Chem Soc. 2026 Oct 15;148(42):45830–9. doi: 10.1021/jacs.6c13587 (PMC13721159; doi:10.1021/jacs.6c13587)
Supplement: Supplementary file 1 [file ja6c13587_si_001.pdf]

Supporting Information

Autonomous biogenesis of early and late assembly intermediates of a large ribosomal subunit in a minimal system

Noa Avidan[1], Michael Levy[2], Yuval Oren[1], Yoshihiro Shimizu[3], Roy H. Bar-Ziv[1*], Shirley S. Daube[1*]

[1] Department of Chemical and Biological Physics, The Weizmann Institute of Science, Rehovot 7610001, Israel

[2] Faculty of Biotechnology and Food Engineering, Technion – Israel Institute of Technology, Haifa, 3200003, Israel

[3] Laboratory for Cell-Free Protein Synthesis, RIKEN Center for Biosystems Dynamics Research, Suita, Osaka 565-0874, Japan

*Corresponding authors

Roy H. Bar-Ziv roy.bar-ziv@weizmann.ac.il

Shirley S. Daube Shirley.daube@weizmann.ac.il

Experimental procedures

Data and statistical analysis

DNA Sequence information

Additional experimental details

Experimental procedures

DNA preparations: All genes coding for proteins were cloned into pIVEX cloning vectors (Roche), which are optimized for protein expression in cell-free systems. Genes coding for each r-protein were inserted into plasmids pIVEX 2.6 under control of the T7 promoter with and without C-terminus hemagglutinin (HA) tags. The 23S and 5S rRNA genes of the rrnB operon were cloned into the pPURE cloning vector (Cosmo Bio USA) to be expressed under the T7 promoter, and an HDV ribozyme [1] was inserted at the 3' end for exact cleavage of each construct. In most 23S constructs, a broccoli aptamer [2] was inserted into helix 9 of the 23S rRNA gene for fluorescent labeling upon transcription, and in one construct it was inserted at the 3' end, to verify that for most transcripts, synthesis in the PURE system of the long rRNA reaches the end. An additional construct which contained both rRNA genes under two T7 promoters with opposite orientation was cloned into pPURE, to ensure stoichiometric presence of the genes coding for the rRNA. All relevant sequences are detailed in Table S1.

Preparation of Linear DNA for DNA Brushes: Linear double stranded DNA was amplified from plasmids containing the relevant constructs using PCR, with the KAPA HiFi HotStart ReadyMix PCR kit (KAPA Biosystems). One primer was modified with 5' biotin, and the other was either plain or fluorescently labelled on the 5' end with the ATTO-647 fluorophore (Integrated DNA Technologies). For all single-gene constructs, the fluorescently labeled or plain forward primer was complementary to a region 200-300 bp upstream of the T7 promoter. In the opposite-orientation rRNA construct, the forward primer was at the end of the 5S gene in a way that enabled its runoff transcription, while the biotinylated reverse primer was complementary to a region downstream to the terminator of the 23S gene. All primer sequences are detailed in Table S2. The DNA was incubated for 30 minutes with streptavidin (Sigma) at a 1.4 SA-DNA ratio in order to create DNA-SA conjugates and diluted to a final concentration of 150nM in 1x phosphate buffered saline (PBS) supplemented with 7% glycerol, the latter added to reduce evaporation at the following DNA surface deposition step.

Photoactivable Biocompatible Monolayer Assembly: Fused-silica slides (24 x 24 x 1 mm, UQG Optics) were coated with a photoactivable biocompatible monolayer according to a previously published protocol [3] . Briefly, the fused-silica slides were cleaned in boiling ethanol for 10 minutes

followed by sonication for 10 minutes and base piranha cleaning (H_2O_2:NH_3:H_2O; 1:1:4, heated to 70°C for 10 minutes). The slides were then coated with a polymer composed of a polyethylene glycol backbone with a protected amine at one end, and a triethoxysilyl group at the other end. The slides were incubated with the polymer solution (0.2 mg/mL in Toluene) for 20 minutes during which the monolayer is formed, and then washed and dried. The coated slides were exposed to 365 nm UV light (2.5 J/cm2) through a custom photomask containing an array of 30 µm hexagons (CAD/Art Services) using UV-KUB (Kloe). Surface amines located inside hexagons were exposed to a saturating UV dose and were fully deprotected, whereas surface amines located between hexagons were partially deprotected due to undesired leaks through the mask. After the UV exposure the slides were immediately covered with biotin N-hydroxysuccinimidyl ester (biotin-NHS, Pierce) dissolved in a borate buffer solution (0.5 mg/mL) and incubated for 30 minutes, during which the biotin bound covalently to the exposed amine groups. The slides were then washed and dried.

Assembly of tape wells with DNA brushes: The biotin-patterned slides were fixed on custom fused silica prisms (Zell Quarzglas und Technische Keramik) with Frame-Seal Slide Chambers adhesive (Bio-Rad). Rectangular or square wells with sides of length ~1.4 cm were cut into adhesive tape of thickness ~50 µm, which was then applied to the biotin-patterned fused silica slide. Nano-liter DNA-SA droplets were individually deposited onto the surface inside the wells using the GIX Microplotter II (Sonoplot Inc., Middleton, WI). The droplets were arranged as an equilateral cluster of 3 brushes (Figure 2 B), or as a cluster of 20 identical brushes (Figure 3 A) to raise the expression levels when expressing all ribosomal components simultaneously, with 120µm between the centers of the droplets. All brush clusters on a single chip were done in the same chamber, at a distance of at least 2mm from each other to prevent crosstalk. The droplets were incubated overnight at a humidity level of 50-65% to allow DNA brush build-up through the biotin-streptavidin linkage. Anti-HA antibodies were patterned on the surface surrounding the DNA brushes, as detailed below. The space between the prism and the slide was filled with index-matching liquid (Cargille) prior to imaging.

Antibody patterning: Biotinylated anti–HA antibodies (50 μg/ml; High Affinity, 3F10 clone, Sigma-Aldrich) were mixed with streptavidin (Sigma) at a molar ratio of 1:1.5 in 1x PBS and incubated

for 30 min on ice, followed by dilution to 50nM in 1x PBS. The solution of SA–anti-HA conjugates was used to wash the surface of the wells containing the DNA-SA droplets and incubated at 4°C for one hour. The surface was washed several times with PBS and then with 50 mM Potassium-HEPES, pH7.4, never being allowed to dry, and was kept in the HEPES buffer until the addition of the reconstituted gene expression reaction mix (PUREfrex2.0, Cosmo Bio USA).

Reconstituted in vitro transcription-translation on chip: Before the experiment, the prism containing the fused-silica slide was positioned on a temperature-controlled holder set at 17°C placed on an upright microscope (Olympus BX51WI). Humidity in the room was reduced to avoid condensation on the prism. The HEPES Potassium buffer in the chamber was exchanged for PUREfrex 2.0 (Cosmo Bio USA), supplemented with DFHBI-1T (Lucerna, NY) at a final concentration of 50µM. Reactions done in assembly conditions were also supplemented with PEG 8000 at a final concentration of 2% or 4% (as indicated for each experiment), GroE mix (Cosmo Bio USA) toward a final concentration of 187nM GroEL and 375nM GroES, DnaK mix (Cosmo Bio USA) toward a final concentration of 1.8µM DnaK, 375nM DnaJ and 375nM GrpE. The chamber was sealed with a glass coverslip, and the temperature was then switched to 37°C to trigger gene expression. Measurement started only after the system reached 37°C.

Total Internal Reflection Fluorescence (TIRF) Microscopy: Total Internal Reflection Fluorescence (TIRF) microscopy was used to image fluorescent molecules close to the surface of the fused-silica slide inside the PDMS wells. The microscope was positioned on a motorized stage (Scientifica). It was equipped with optical filter sets for excitation at 488 and 647 nm and a fluorescent light source (EXFO X-Cite 120Q) to allow epifluorescence microscopy. Two-color TIRF microscopy was achieved by combining and coupling two lasers (OBIS 488-150 LS and OBIS 647 LX, Coherent) into a single-mode optical fiber (Oz optics). The beam was then collimated and directed on the prism using a goniometer (Thorlabs) to reach the surface of the chamber at an angle of total internal reflection, inducing an evanescent wave on the surface of the chamber. Epifluorescence and TIRF images were taken with Andor iXon Ultra camera (Andor Technology plc., Belfast, UK) and a 10X Olympus objective. The stage, the microscope, the lasers and the camera were controlled through LabVIEW (National Instruments). Images were taken of the DNA brush clusters using the TIRF microscope, centered on the brushes themselves (see Figure 1 B).

r-protein-HA expression in silicon compartments: The gene constructs coding for the HA-tagged r-proteins were diluted in noncoding DNA at a 1:9 ratio and immobilized on the surface of compartments 400µm in diameter on a silicon chip according to a previously published protocol [4] . PUREfrex 2.0 (Cosmo Bio USA) was applied to the chip, and the resulting r-proteins were captured by anti-HA antibodies bound to the surface of the chamber. After 1 hour of expression at 37°C, the chip was washed with pre-expressed GFP-HA, and fluorescence was measured using a fluorescence microscope, such that the fluorescence signal represents a negative measurement. The average fluorescence signal of a blank well was subtracted from the resulting fluorescence signal in each well and normalized by the average fluorescence signal of a blank well.

In vitro transcription in silicon compartments: Linear DNA fragments coding for the H9 or 3'-Fl-23S rRNA were immobilized on the surface of silicon compartments 400µm in diameter according to a previously published protocol [4] . A solution of PUREfrex 2.0 (Cosmo Bio USA) was mixed according to the manufacturer's instructions, except that sol III (ribosomes) was omitted and replaced with water. The solution was supplemented with 10µM DFHBI (Lucerna, NY), applied on the chip and the chip was sealed with a PDMS slice. After 1 hour of incubation at 37°C the fluorescence in each compartment was measured using a fluorescence microscope without opening the PDMS cover.

Gel electrophoresis analysis of in vitro transcribed rRNA. HiScribe® T7 High Yield RNA Synthesis reactions (New England Biolabs) were mixed according to the manufacturer's instructions. Each 10ul reaction was supplemented with ~30nM of linear DNA corresponding to each of the tested rRNA constructs. The reactions were incubated for 2-3 hours at 37°C and quenched with SDS load buffer (at a final concentration of 2% SDS, 10% glycerol, 5% 2-mercaptoethanol, 0.002% bromphenol blue and 62.5 mM Tris HCl, pH 6.8). 2ul of each reaction were loaded on a 2.5% agarose gel containing 1× Tris-acetate-EDTA (TAE) running buffer (pH ~ 8.0) prepared from a 50× stock solution (BioLab). After gel electrophoresis at 100V the gel was washed twice for 3 minutes with water and then stained for 10–30 min in 10 µM DFHBI-1T (Lucerna) in 40 mM HEPES pH 7.4, 100 mM KCl, 1 mM $MgCl_2$ [5]. The gel was scanned using FLA-5100 scanner (FUJIFILM) using 488 and 532 lasers. The Broccoli aptamer signal (detected with a 488 laser) could not be distinguished

from the ethidium bromide fluorescence signal (detected with both 488 and 532 lasers) most likely due to contaminating ethidium bromide in the gel apparatus.

In vitro transcription-translation bulk reactions for testing protein expression: Solutions of PUREfrex 2.0 (Cosmo Bio USA) were mixed according to the manufacturer's instructions and supplemented with 3nM plasmid DNA encoding the desired proteins. The reactions were incubated for 2-3 hours at 37°C and quenched with SDS load buffer (at a final concentration of 2% SDS, 10% glycerol, 5% 2-mercaptoethanol, 0.002% bromphenol blue and 62.5 mM Tris HCl, pH 6.8. The nascent proteins were resolved via gel electrophoresis in mPAGE® 4-20% Bis-Tris Precast Gels (Millipore), under the conditions recommended by the manufacturer. The nascent proteins were detected in two ways. For in situ labeling the PURE reactions were supplemented with 4% Green Lys In Vitro Translation Labelling System ($green_{Lys}$, FluoroTect, Promega) and after incubation and quenching a sample was loaded directly on the mPAGE gel. To monitor expression independent of in situ labeling, proteins with an HA tagged were expressed in the PURE system and resolved by the mPAGE, as described above. Following electrophoresis, proteins were electrophoretically transferred to a mini-size LF PVDF membrane (Bio-Rad Laboratories) using the Trans-Blot Turbo Transfer System (Bio-Rad Laboratories) according to the manufacturer's instructions using the pre-programmed Standard protocol (constant 1.0 A, up to 25 V) for 30 minutes. Following transfer, the membrane was immediately processed for immunoblotting, beginning with blocking, followed by incubation with 1:5000 dilution of HA-probe Antibody (F-7) Alexa Fluor® 647 (sc-7392 AF647, Santa Cruz). The membrane was scanned using FLA-5100 scanner (FUJIFILM).

Data Analysis: Images obtained from the microscope underwent basic analysis in ImageJ, used to average pixel intensity over selections in image stacks. For each brush cluster, the hexagon signal was measured as the average signal over five hexagons chosen near the center of the image where there was no DNA, with local background (the average signal over small areas adjacent to the chosen hexagons) subtracted. Further analysis was done in Python. The kinetic scatter plots show the background-subtracted hexagon signal on the y axis. The timepoints for the x axis were obtained using the timestamps on the microscopy images, with t=0 defined as

the timepoint at which the system reached 30°C when the chip temperature was raised from 17°C to 37°C. To further analyze the kinetic curves, we applied a Savitsky-Golay filter with smoothing bin 8 and polynomial fit of order 1 to each time-signal curve to smooth the data (using the function scipy.signal.savgol_filter). The max fluorescence was calculated for each brush cluster over the first 100 minutes of the measurement, and averaged across all replicas for the bar plots.

Plotting average dynamic curves: The Savitsky-Golay smoothed data was interpolated onto evenly spaced timepoints to make the x axis data line up, and the average signal and its standard deviation over the replicas was found per timepoint and plotted. Example code for interpolation:

```
import numpy as np
xdata_range_for_mean_sig = np.arange(start=10, stop=95, step = 2)
ydata_savgol_interpolated = np.interp(xdata_range_for_mean_sig, xdata, ydata_savgol)
```

Note that the average of the maximum values plotted in the bar plots does not correspond to the maximum of the average dynamics.

Calculating the onset time: Each curve in the 20-brush reactions (Figure 4, Figure S7) was defined as “on” if its spread (the difference between max and min signal) was above a certain threshold corresponding to the signal for which it was possible to see the hexagonal pattern by eye (70). For the curves that turned on, the onset time was calculated as the time when three adjacent points rose above a slightly lower threshold (50), corresponding to the beginning of a clear rise in the signal. We chose to find the average onset time only for the r-proteins that gave an “on” signal for all replicas, as well as three r-proteins (uL1, bL19 and bL34) that gave an “on” signal for 5 out of 6 replicas (all turned on except 1), since we considered more than one replica being flat (“off”) as an inconsistent signal that cannot have an average onset time. On the plot, the dashed line corresponds to the start of the measurement. Onset times close to the dashed line may be earlier than what is shown.

Statistical analysis: For comparison between two different conditions (the data shown in Figure 3E, and Figure 6C, E), Student t-tests were used, with the equal variance condition assumed false as the noise is assumed to be dependent on signal strength. Differences in signal were considered significant with p value below 5%. Due to the low number of replicas, the result of a permutation

test is shown as well. To show that an increase in ObgE and EngA concentration results in an increase of binding signal for uL16 and bL28 (Figure 5E), the Spearman test was used to prove monotonic increase. The results of all the statistical tests are shown in Table S3. The relevant Python functions:

```
Student t-test:
t_stat, p_val = scipy.stats.ttest_ind(sample1, sample2, equal_var=False)
Permutation test:
res_permutation = scipy.stats.permutation_test((sample1, sample2), statistic_permute_mean, permutation_type='independent')
Spearman test (using pandas dataframe df containing the max signal of each replica):
rho, p_value = scipy.stats.spearmanr(df['Factor amount'], df['Max signal'])
```

DNA sequences for surface-based LSU biogenesis assay:

Description	Sequence
**r-protein gene without HA.**<br>Blue: T7 promoter.<br>Green: UTR1.<br>Orange: r-protein gene (bL36 presented as example).<br>Red: T7 terminator.	TAATACGACTCACTATAGGGAGACCACAACGGTTTCCCTCTAG<br>AAATAATTTTGTTTAACTTTAAGAAGGAGATATACCATGAAAG<br>TTCGTGCTTCCGTCAAGAAATTATGCCGTAACTGCAAAATCGT<br>TAAGCGTGATGGTGTCATCCGTGTGATTTGCAGTGCCGAGCC<br>GAAGCATAAACAGCGCCAAGGCTGACATATGCCCGGGCTCG<br>AGGGACCCCGCGGGCGGCCGCGTCGACGGATCCGGCTGCTA<br>ACAAAGCCCGAAAGGAAGCTGAGTTGGCTGCTGCCACCGCTG<br>AGCAATAACTAGCATAACCCCTTGGGGCCTCTAAACGGGTCTT<br>GAGGGGTTTTTTG
**C-terminus HA tag.**<br>Orange: end of r-protein gene (bL36 presented as example).<br>Grey: Linker.<br>Violet: HA tag.<br>Black: Stop codon.	…GTCATCCGTGTGATTTGCAGTGCCGAGCCGAAGCATAAACA<br>GCGCCAAGGCAAGCGAGCTCCCGGGACCAGCTACCCATACG<br>ATGTTCCAGATTACGCTTAA
**Assembly factor gene.**<br>Blue: T7 promoter.<br>Green: UTR1.<br>Orange: assembly factor gene (DeaD presented as example).<br>Red: T7 terminator.	TAATACGACTCACTATAGGGAGACCACAACGGTTTCCCTCTAG<br>AAATAATTTTGTTTAACTTTAAGAAGGAGATATACCATGGCTG<br>AATTCGAAACCACTTTT…CGGTGGTGATGCGTAACTAACTAA<br>GATCCGGTAAGATCCGGCTGCTAACAAAGCCCGAAAGGAAG<br>CTGAGTTGGCTGCTGCCACCGCTGAGCAATAACTAGCATAAC<br>CCCTTGGGGCCTCTAAACGGGTCTTGAGGGGTTTTTTGCTGA<br>AAGGAGGAACTATA…
**5S construct.**<br>Blue: T7 promoter.<br>Orange: 5S gene of the rrnB operon.<br>Brown: HDV sequence.<br>Red: T7 terminator.	TAATACGACTCACTATAGTGCCTGGCGGCAGTAGCGCGGTGG<br>TCCCACCTGACCCCATGCCGAACTCAGAAGTGAAACGCCGTA<br>GCGCCGATGGTAGTGTGGGGTCTCCCCATGCGAGAGTAGGG<br>AACTGCCAGGCATGCCGGCCATGGTCCCAGCCTCCTCGCTGG<br>CGGCCGGTGGGCAACATTCCGAGGGGACCGTCCCCTCGGTAA<br>TGGCGAATGGGACGTAACTAGCATAACCCCTTGGGGCCTCTA<br>AACGGGTCTTGAGGGGTTTTTTG
**23S construct.**<br>Blue: T7 promoter.<br>Orange: 23S gene.<br>Brown: HDV.<br>Red: T7 terminator.	TAATACGACTCACTATAGGTTAAGCGACTAAGCGTACACGGT<br>GGATGC…AACCGTGAGGCTTAACCTTGCCGGCCATGGTCCCA<br>GCCTCCTCGCTGGCGGCCGGTGGGCAACATTCCGAGGGGAC<br>CGTCCCCTCGGTAATGGCGAATGGGACGTAACTAGCATAACC<br>CCTTGGGGCCTCTAAACGGGTCTTGAGGGGTTTTTTG

**23S H9 broccoli.**<br>Orange: 23S gene.<br>Green: broccoli aptamer.	…GAAACCGTGTACGTACAAGCAGTGGGAGCACGCTTGCGGA<br>GACGGTCGGGTCCAGATATTCGTATCTGTCGAGTAGAGTGTG<br>GGCTCCGCAGGCGTGTGACTGCGTACCTTTTGTATAATGGGT<br>CAGC…
**23S 3' broccoli.**<br>Orange: 23S gene.<br>Green: broccoli aptamer.<br>Brown: HDV.	…AATGAACCGTGAGGCTTAACCTTGCGGAGACGGTCGGGTC<br>CAGATATTCGTATCTGTCGAGTAGAGTGTGGGCTCCGCGCCG<br>GCCATGGTCCCAGCCTCCTCGCTGGCGGCCGGTGGGCAACAT<br>TCCGAGGGGACCGTCCCCTCGGTAATGGCGAATGGGAC
**Back-to-back 23S-H9FL-5S construct.**<br>Orange: backward 5S gene of the rrnB operon.<br>Light blue: backward T7 promoter.<br>Blue: T7 promoter.<br>Yellow: 23S gene of the rrnB operon.<br>Green: broccoli aptamer.<br>Brown: HDV sequence.<br>Red: T7 terminator.<br>Black: sequences in the pPURE plasmid.	…AATAAACAAATAGGGGTTCCGCGATGCCTGGCAGTTCCCTA<br>CTCTCGCATGGGGAGACCCCACACTACCATCGGCGCTACGGC<br>GTTTCACTTCTGAGTTCGGCATGGGGTCAGGTGGGACCACCG<br>CGCTACTGCCGCCAGGCACTATAGTGAGTCGTATTACACATTT<br>CCCCGAAAAGTGCTAGTGGTGCTAGCCCCGCGAAATTAATAC<br>GACTCACTATAGGTTAAGCGACTAAGCGTACACGGTGGATGC<br>CCTGGCAGTCAGAGGCGATGAAGGACGTGCTAATCTGCGATA<br>AGCGTCGGTAAGGTGATATGAACCGTTATAACCGGCGATTTC<br>CGAATGGGGAAACCCAGTGTGTTTGCGGAGACGGTCGGGTC<br>CAGATATTCGTATCTGTCGAGTAGAGTGTGGGCTCCGCCGAC<br>ACACTATCATTAACTGAATCC…GTACTAATGAACCGTGAGGCT<br>TAACCTTGCCGGCCATGGTCCCAGCCTCCTCGCTGGCGGCCG<br>GTGGGCAACATTCCGAGGGGACCGTCCCCTCGGTAATGGCGA<br>ATGGGACGTAACTAGCATAACCCCTTGGGGCCTCTAAACGGG<br>TCTTGAGGGGTTTTTTGCTGAAAGGAGGAACT…

Table S1: DNA constructs. The constructs coding for the r-proteins were cloned into the pIVEX 2.6 vector, and those coding for the rRNA into the pPURE vector.

**Construct**	**Forward primer**	**Reverse primer (5' biotin)**
23S (broccoli tagged)	AGTTGGCCGCAGTGTTATCAC (5' ATTO647)	CCTTTGAGTGAGCTGATACC (5' biotin)
5S	GTTGGCCGCAGTGTTATCAC	TTGCCGGATCAAGAGCTACC (5' biotin)
Back-to-back H9-FL-23S and 5S genes, with 5S runoff transcription	ATGCCTGGCAGTTCCCTAC (5' ATTO647)	CCTTTGAGTGAGCTGATACC (5' biotin)
**r-proteins**: uL1, uL2, uL3, uL4, uL5, uL6, bL9, uL10 **r-proteins-HA fusions**: uL1-HA, uL2-HA, uL3-HA, uL4-HA, uL5-HA, uL6-HA, bL9-HA, uL10-HA	GATGTGCTGCAAGGCGATTA AG	GCGGTAGTTTATCACAGTTAA ATTGC (5' biotin)
**r-proteins**: bL9, uL11, bL12, uL13, uL14, uL15, uL16, bL17, uL18, bL19, bL20, bL21, uL22, uL23, uL24, bL25, bL27, bL28, uL29, uL30, bL31, bL32, bL33, bL34, bL35, bL36 **r-proteins-HA fusions**: bL9-HA, uL11-HA, bL12-HA, uL13-HA, uL14-HA, uL15-HA, uL16-HA, bL17-HA, uL18-HA, bL19-HA, bL20-HA, bL21-HA, uL22-HA, uL23-HA, uL24-HA, bL25-HA, bL27-HA, bL28-HA, uL29-HA, uL30-HA, bL31-HA, bL32-HA,	GTCTGTAAGCGGATGCCGG	CAATACGCAAACCGCCTCT (5' biotin)

bL33-HA, bL34-HA, bL35-HA, bL36-HA		
**Assembly factor genes**: EngA, ObgE, DbpA, DeaD, SrmB, YhbY	ATGTGCTGCAAGGCGATTAAG (5' ATTO647)	GATCATGGCGACCACACCCGTC (5' biotin)
Noncoding DNA (amplified from pIVEX vector containing gp7 gene of T4 phage [4])	TTTCCTCGTGAATGGTTAGC	GATCATGGCGACCACACCCGTC (5' biotin)

Table S2: Primers for amplification of linear DNA for DNA brush. All primers attach to the vector backbones.

**2-body vs. N-body experiment (Figure 3E)**					
rP-HA	t-score	t-test p-value	Difference between means	Permutation test p-value	
L3	1.09	31.7%	331	31.4%	
L24	0.125	90.6%	86.2	97.1%	
L21	-3.79	2.96%	-1520	2.86%	
L22	-3.45	0.747%	-225	0.38%	
L6	0.277	79.4%	2.97	91.4%	
L16	0.805	45.8%	17.7	62.9%	
**Factor titration experiment (Figure 5E)**					
rP-HA	Spearman's rho	p-value for Spearman			
L16	0.748	$1.61*10^{-5}$%			
L28	0.493	0.224%			
**Factor experiment – 5 more rPs (Figure 6C)**					
rP-HA	t-score	t-test p-value	Difference between means	Permutation test p-value	
L6	-3.43	4.10%	-324	2.86%	
L9	-3.14	4.41%	-199	2.86%	
L22	-0.462	65.8%	-39.2	61.9%	
L32	-6.14	0.104%	-98.1	2.86%	
L36	-2.72	4.58%	-68.0	2.86%	
**Factor experiment – 4 more factors (Figure 6E)**					
rP-HA	Factor set	t-score	t-test p-value	Difference between means	Permutation test p-value
L14	G	-8.10	0.292%	-142	2.86%
	H	-2.12	12.3%	-162	2.86%
L16	G	-3.94	2.19%	-288	2.86%
	H	-5.69	0.527%	-327	2.86%
L21	G	-0.184	85.9%	-25.4	85.7%
	H	-1.22	26.8%	-238	25.7%

Table S3: Statistical analysis for the specified experiments.

Green rows denote statistically significant under both tests (p-value under 5%).

Yellow rows denote statistically significant only under the permutation test (p-value under 5%).

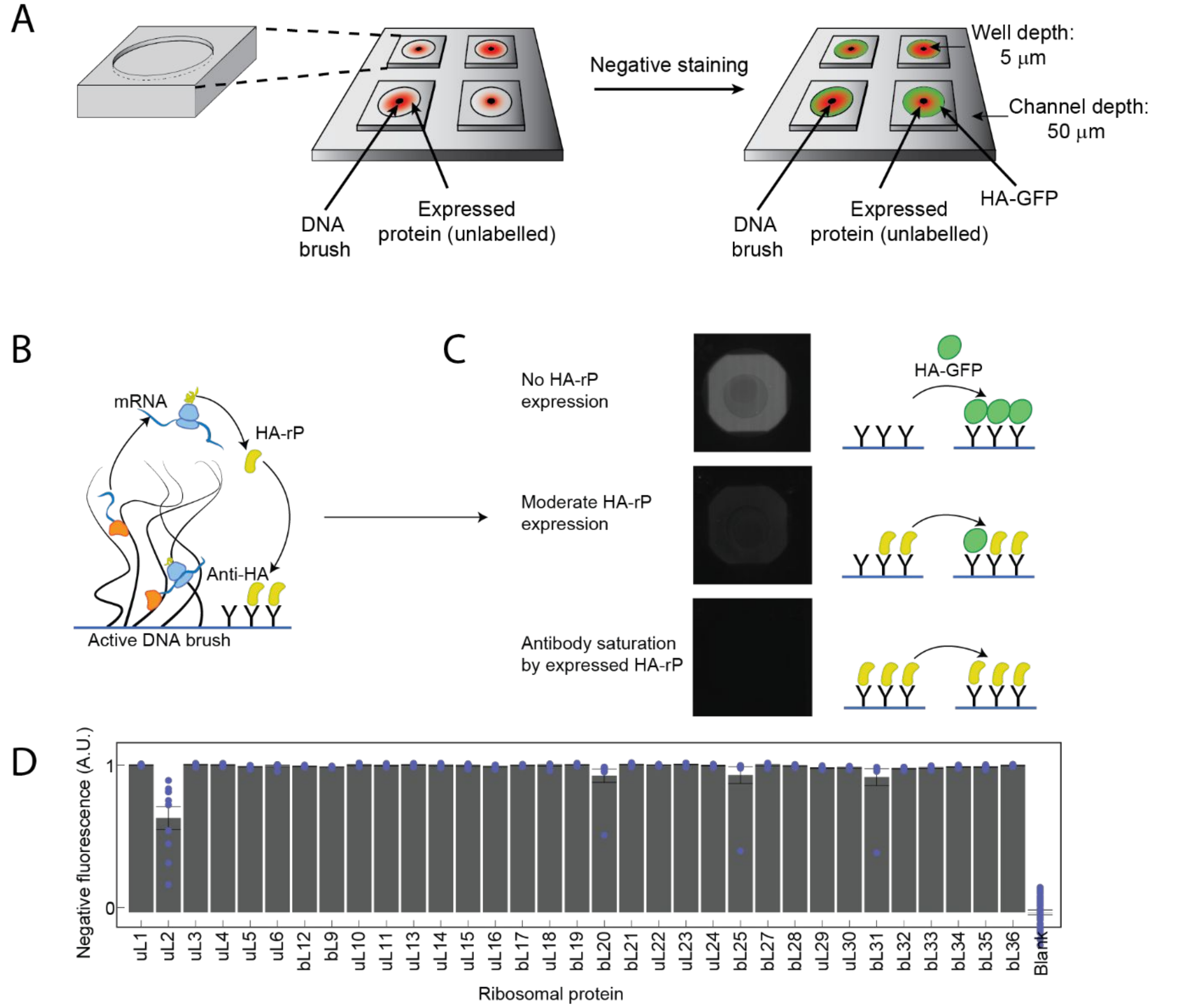

Figure S1: HA-tagged r-protein expression in PURE. (A) DNA brushes containing a gene coding for one HA-tagged r-protein were immobilized in 5 μm deep compartments of 0.4mm in diameter, surrounded by 50 μm deep channels on a silicon biochip. Each r-protein-HA was expressed from its coding gene in PUREfrex 2.0 and bound to surface-immobilized anti-HA antibodies. The wells then underwent negative staining with HA-tagged GFP binding to vacant anti-HA antibodies. The fluorescence signal on the surface was imaged by fluorescence microscopy with 488nm excitation. (B) A scheme showing expression of the r-protein-HA and its binding to the antibodies. (C) Microscopy images of GFP-HA fluorescence in compartments with no r-protein-HA expression (top), r-protein-HA expression that does not saturate the surface (center) and r-protein-HA expression that saturates the surface antibodies (bottom). (D) Normalized negative fluorescence signal representing the occupancy of antibodies on the surface for each HA-tagged r-protein. All r-proteins are expressed and saturate the antibodies on the surface of the wells, except for uL2 which was expressed but did not saturate the surface.

1

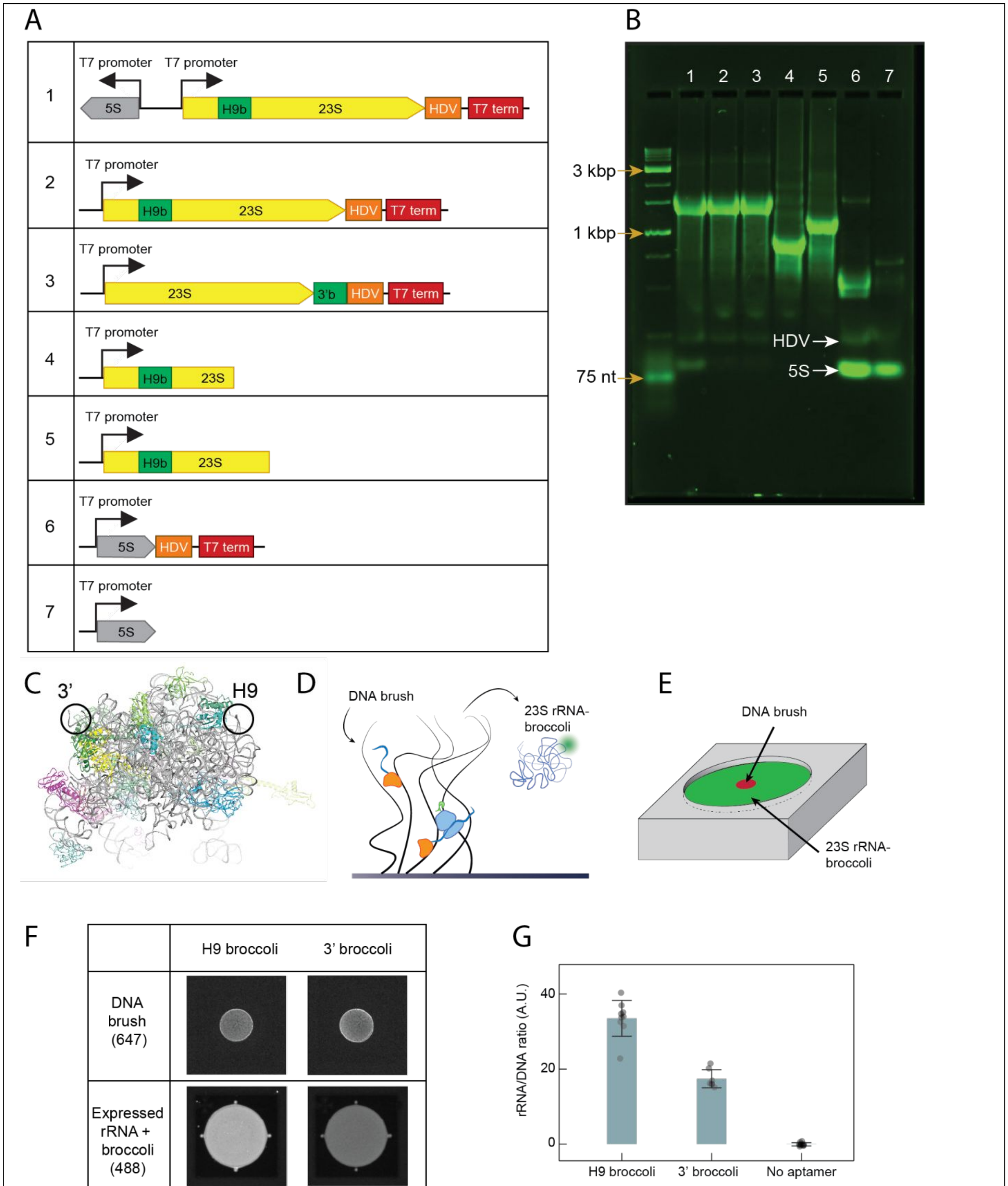

Figure S2: 23S rRNA synthesis. (A) PCR constructs coding for rRNA genes. 1, back-to-back H9FL-23S-HDV/5S-runoff; 2, H9FL-23S-HDV; 3, 3'FL-23S-HDV; 4, H9FL-23S-1500 runoff; 5, H9FL-23S-2100 runoff; 6, 5S-HDV; 5S runoff. Expected lengths of RNA: 1, 23S rRNA 2958nt, HDV 135nt, 5S

120nt; 2, 23S rRNA 2958nt, HDV 135nt; 3, 23S rRNA 2958nt, HDV 135nt; 4, 23S-1500 1478nt; 5, 23S-2100, 2112nt; 6, 5S 120nt, HDV 135nt; 7, 5S 120nt. (B) In vitro transcribed 23S and 5S rRNA from PCR constructs in (A) using HiScribe® T7 High Yield RNA Synthesis reactions (New England Biolabs) were resolved by 2.5% agarose gel and scanned using a 532nm laser, detecting residual ethidium bromide in the gel apparatus (Supporting Information). Arrows on the left size of the gel point to double stranded DNA molecular weight markers. Another arrow points to a single stranded DNA oligo 75 nucleotides long fluorescently labeled with Atto488 which was loaded with the DNA molecular weight markers. (C) The 3D structure of the 50S subunit (PDBID 4V9D) in a state of protein translation, with helix H9 and the 3' end circled. (D) Scheme of a DNA brush coding for 23S rRNA with an embedded broccoli aptamer. In the absence of genes coding for HA-tagged r-proteins, the rRNA remains in solution above the surface. (E) Scheme of a silicon compartment with immobilized DNA brush encoding the broccoli modified rRNA that is filling the compartment upon synthesis from the brush. (F) Fluorescent images of two compartments expressing either H9-23S or 3'-23S rRNA, respectively. The 647 Fl-DNA is detected as a circular DNA brush (top images) and the 488 broccoli signal fills the compartments upon rRNA synthesis in the PURE system, lacking ribosomes (bottom images). (G) Average broccoli fluorescence signal, normalized by the DNA fluorescence signal in each compartment encoding either the H9 or 3' Fl 23S rRNA. The control compartments included DNA brushes encoding GFP. In the absence of ribosomes, GFP is not synthesized and the mRNA encoding it does not include a broccoli aptamer, serving as a negative control.

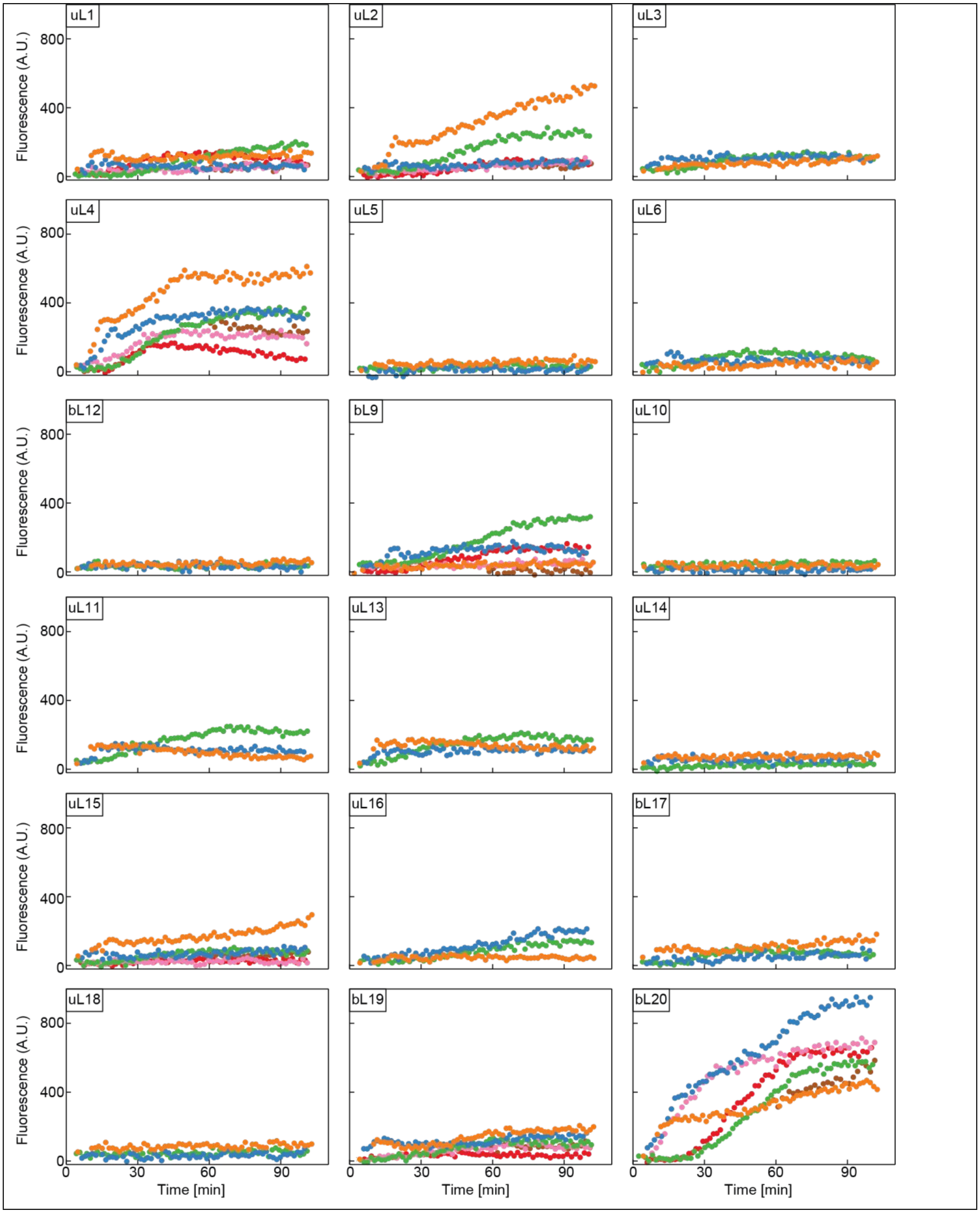

uL1
uL2
uL3
uL4
uL5
uL6
bL12
bL9
uL10
uL11
uL13
uL14
uL15
uL16
bL17
uL18
bL19
bL20
Fluorescence (A.U.)
800
400
0
30
60
90
Time [min]

1

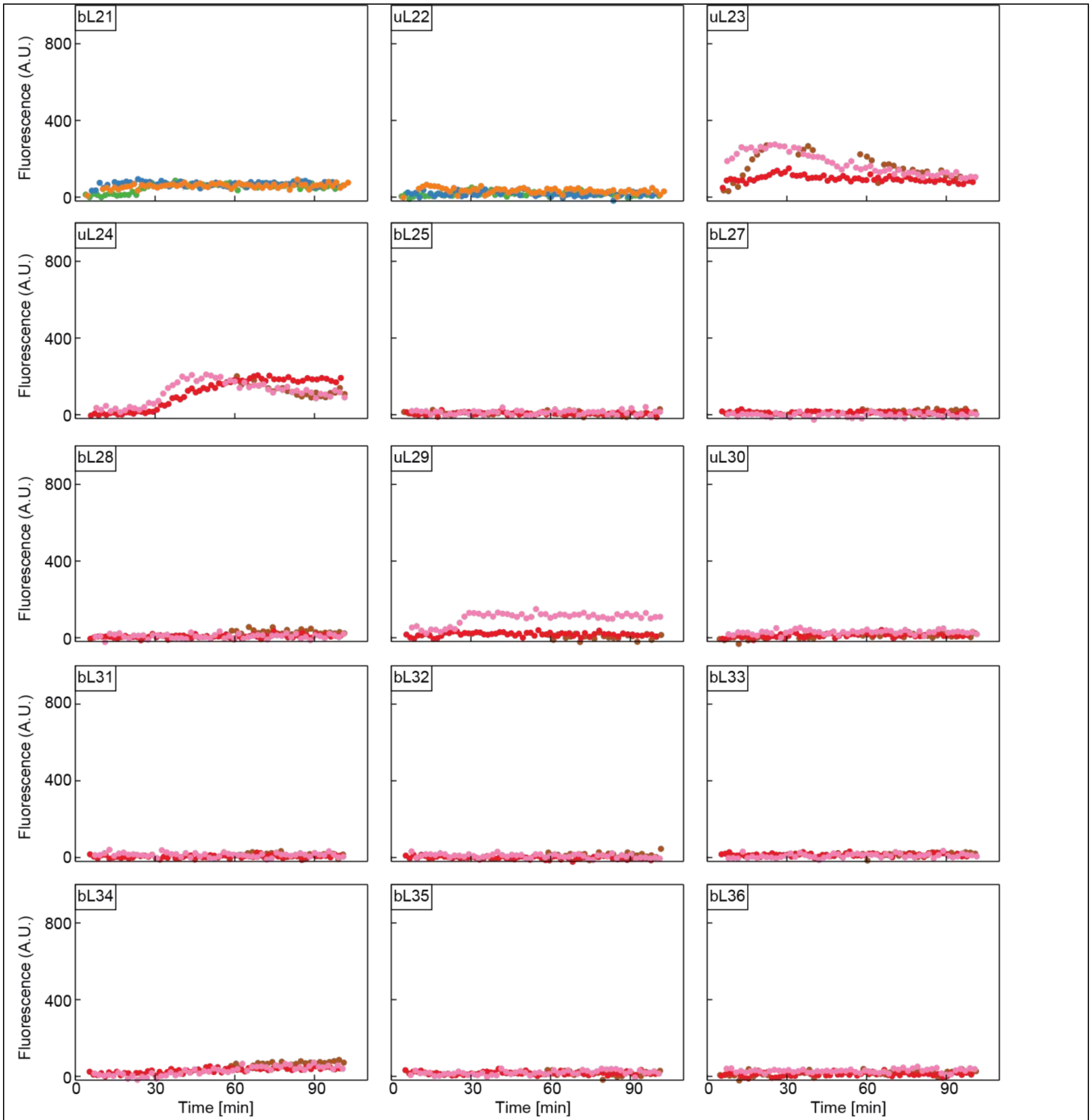

Figure S3: Coupled synthesis and interaction of each LSU r-protein with the 23S rRNA. All kinetic curves of the data shown in Figure 2E, G. Each panel contains the dynamics of replicas of the same DNA brush setup. Plots of the same color represent clusters from the same reaction chamber. The interaction of the FL-rRNA with each HA-tagged r-protein was repeated at least 3 times.

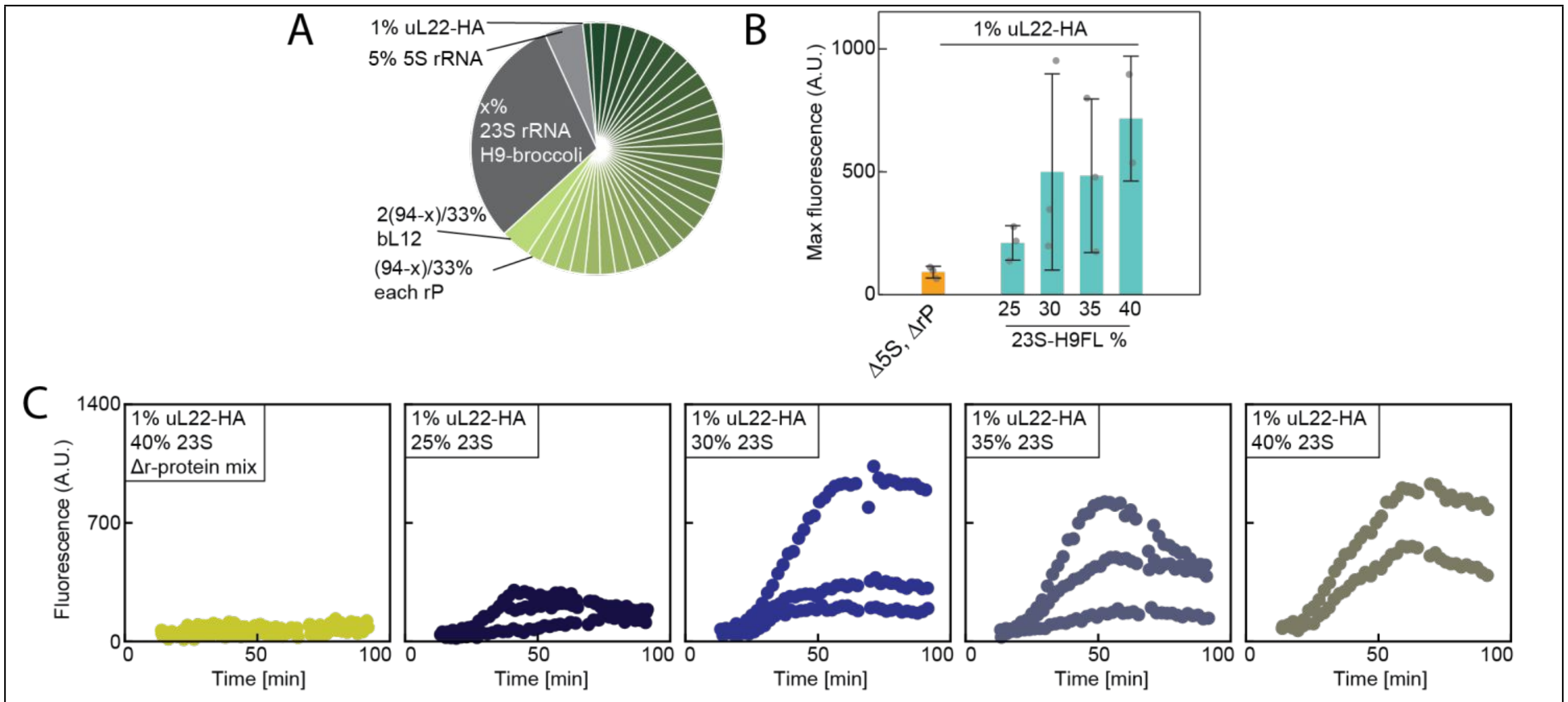

Figure S4: Gene ratio calibration for uL22-HA binding to H9-FL-23S in the presence of genes coding for all ribosomal components. (A) For gene ratio calibration, DNA mixes were spotted on the well surface in a 20-brush configuration (see Figure 3A) with the gene ratio: x% H9-FL-23S, 5% 5S rRNA, 1% uL22-HA, and (94-x)% an equal mix of the other r-proteins, where x is between 25 and 40. (B) The max signal over 100 minutes of uL22-FL-23S binding for the different gene ratios detailed in (A), across 2-3 repeats, and its standard deviation. (C) All kinetic curves of the data shown in (B). Each panel contains the dynamics of replicas of the same DNA brush setup.

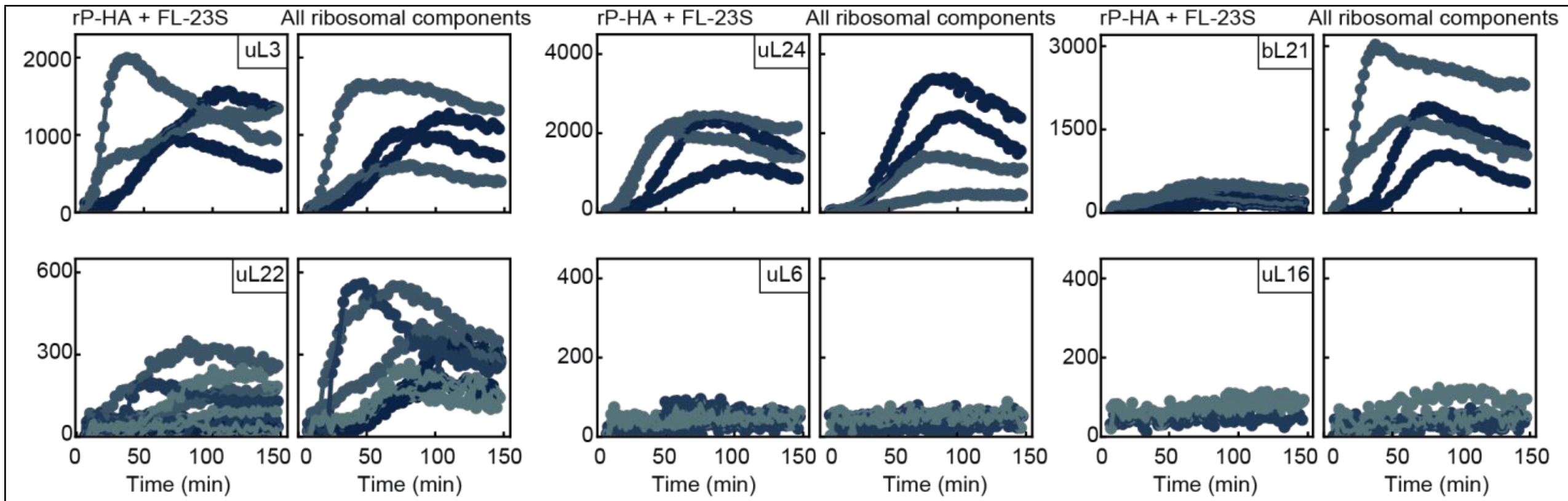

Figure S5: Synthesis and assembly of several r-proteins-HA to the 23S under assembly conditions, in the presence and absence of genes coding for the other ribosomal components. All kinetic curves of the data shown in Figure 3D, E. Each panel contains the dynamics of replicas of the same DNA brush setup. Plots of the same color represent clusters from the same reaction chamber.

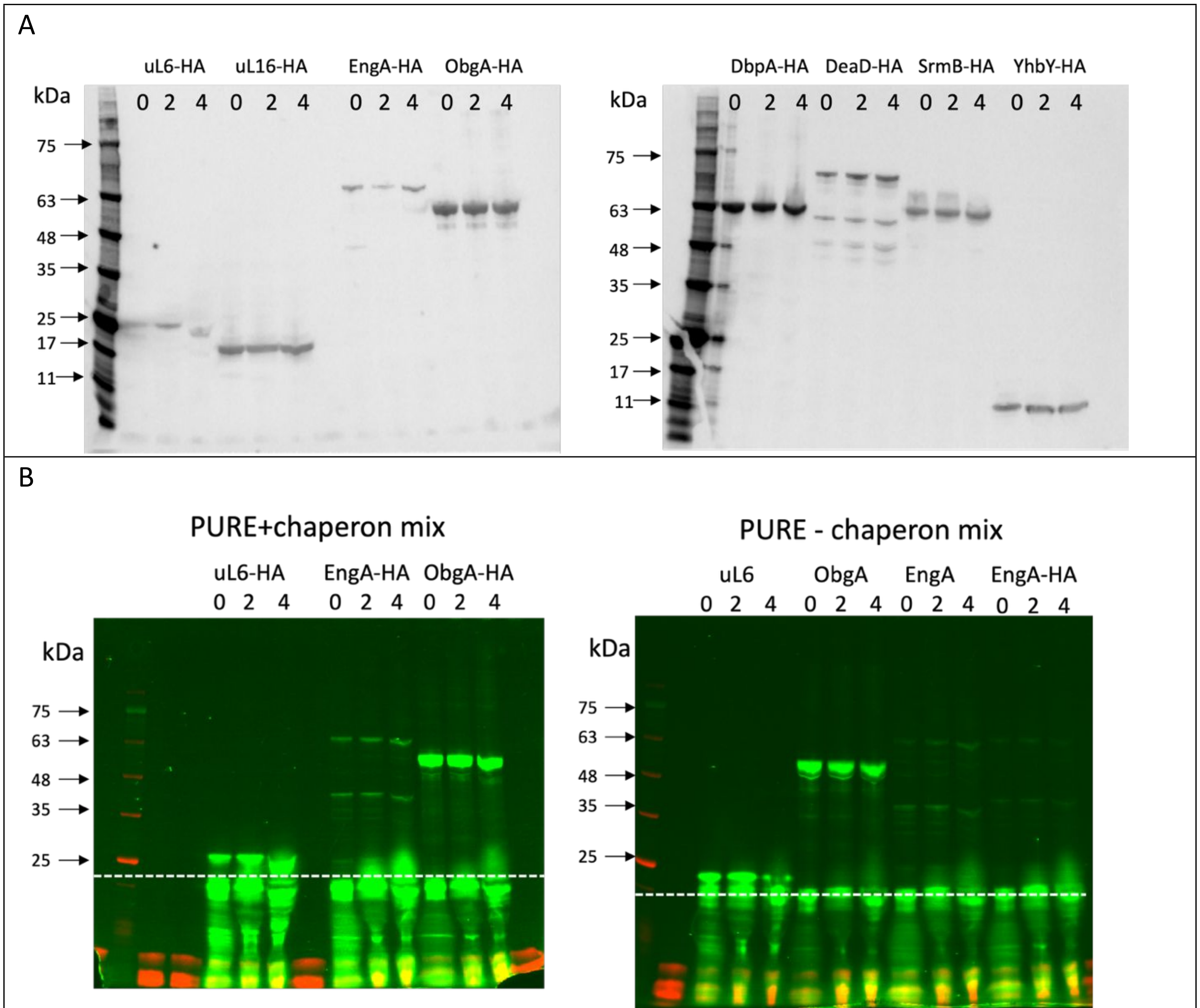

Figure S6: Protein expression in the PURE system at different PEG concentrations. (A) Two r-proteins (uL6 20.6kDa and uL16 17.0kDa) and six assembly factors (EngA 56.8 kDa, ObgE 45.0 kDa, DbpA 49.1 kDa, DeaD 72.3 kDa, SrmB 51.6 kDa, YhbY 10.7 kDa) were expressed in PURE reactions supplemented with either 2%, 4% or no PEG, as indicated at the top of the gels. All the proteins included an HA peptide tag at their C terminus. Proteins were resolved by 4-20% SDS-PAGE, and transferred to a PVDF membrane that was blotted with anti-HA antibodies (Supporting Information). Molecular Weight Markers are indicated on the left side of each gel The proteins vary by their expression levels but overall their expression is not affected by the PEG concentration. (B) Two r-proteins (uL6 20.6 kDa and uL16 17.0 kDa) and two assembly factors (EngA 56.8 kDa, ObgE 45.0 kDa) were expressed in PURE reactions supplemented with $green_{Lys}$ and either with (left gel) or without (right gel) protein chaperons (Supporting Information). PEG 8000 concentration is indicated at the top of the gels. Proteins were resolved by 4-20% SDS-PAGE and imaged directly (Supporting Information). Molecular Weight Markers are indicated on the left side of each gel in kDa. All bands in the gels below the white dashed line are related to the $green_{Lys}$ reagent. No effect of PEG was observed on protein expression

levels in the PURE reactions. Taken together with the western blot analysis in (A), neither the HA tag nor the $green_{Lys}$ reagent affected the expression level.

1

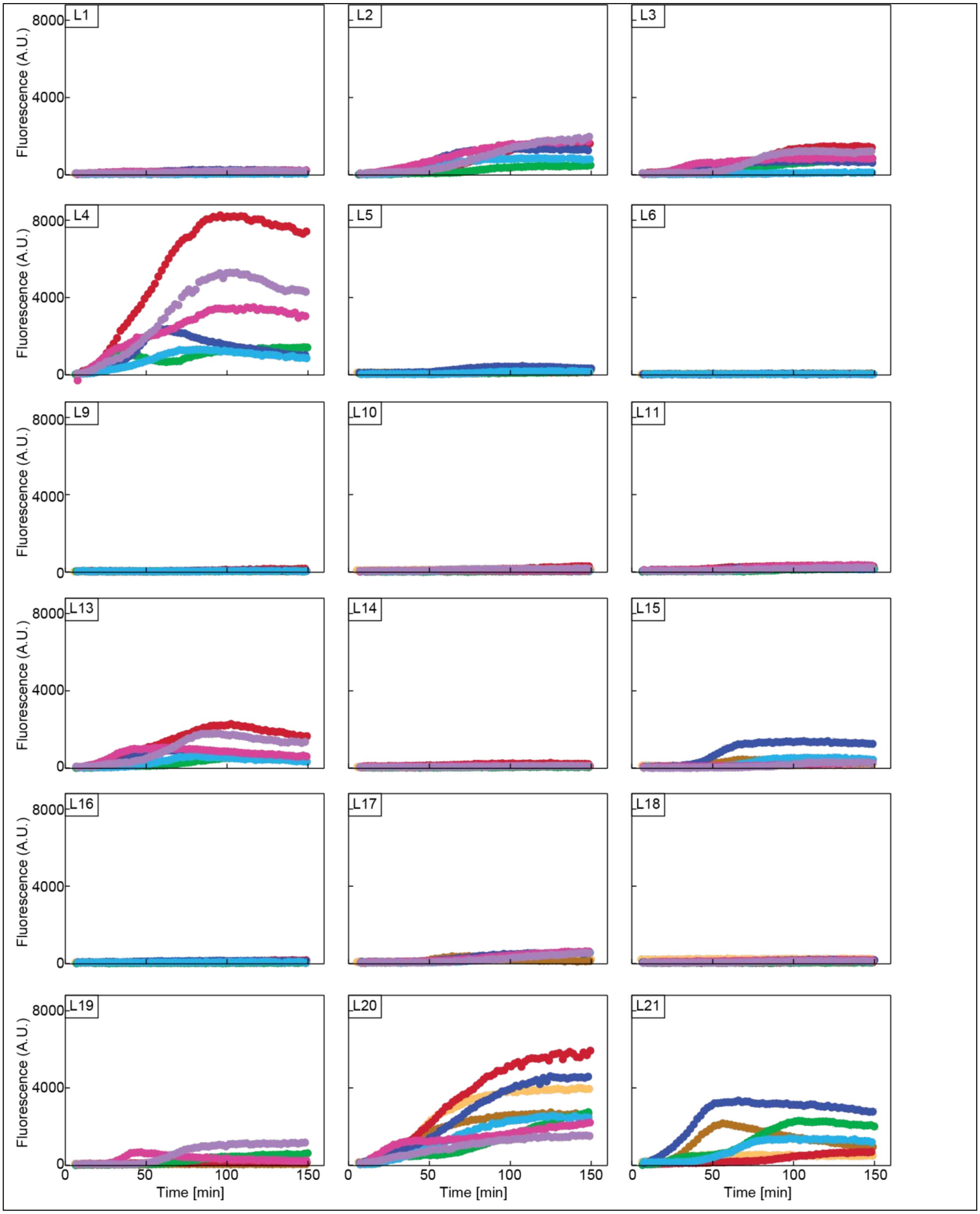
L1
L2
L3
L4
L5
L6
L9
L10
L11
L13
L14
L15
L16
L17
L18
L19
L20
L21
Fluorescence (A.U.)
8000
4000
0
0
50
100
150
Time [min]

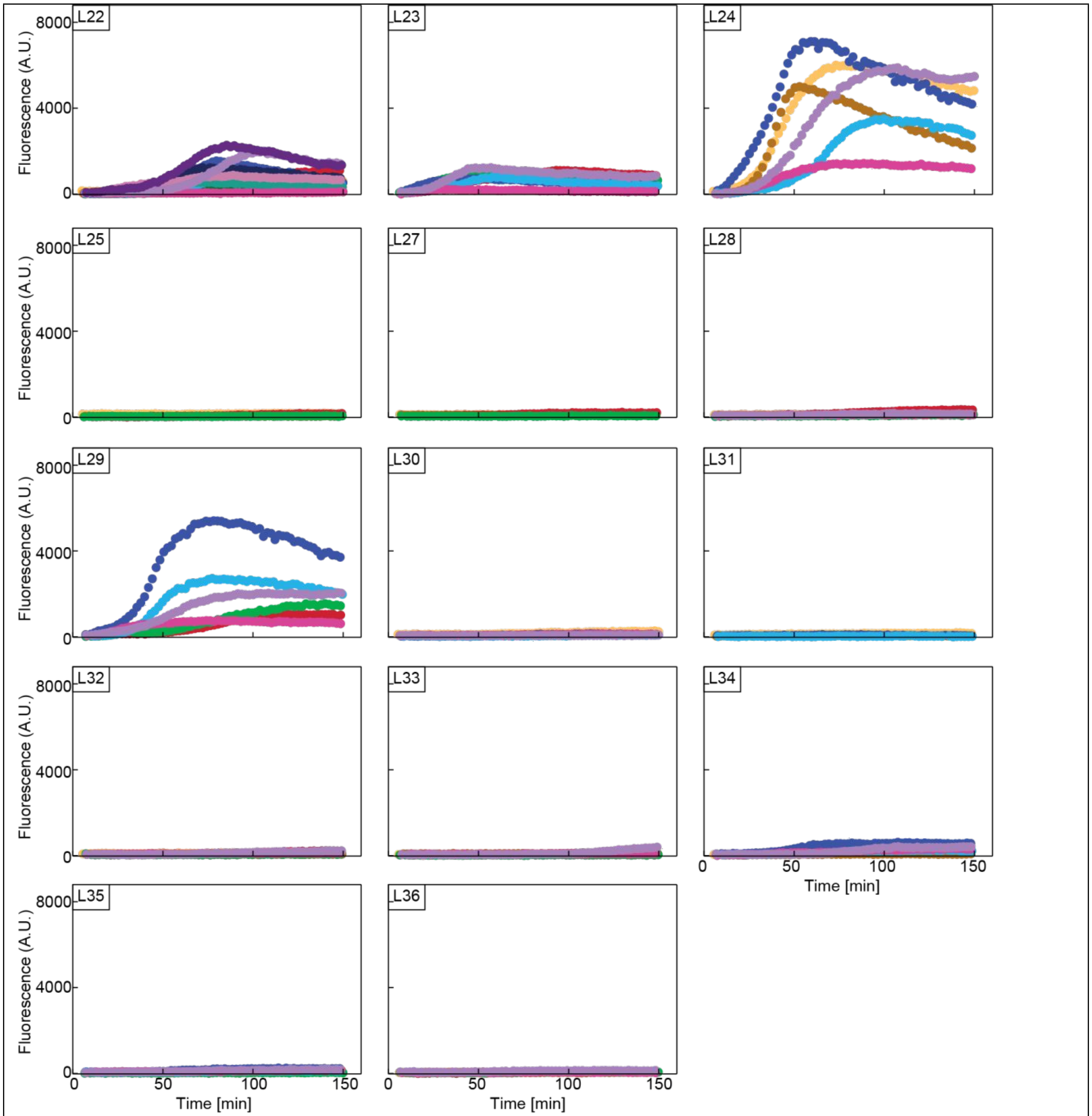

Figure S7: Coupled synthesis and assembly of all LSU components. All kinetic curves of the data shown in Figure 4A, B. Each panel contains the dynamics of replicas of the same DNA brush setup. Plots of the same color represent clusters from the same reaction chamber. The interaction of the FL-rRNA with each HA-tagged r-protein was repeated at least 4 times.

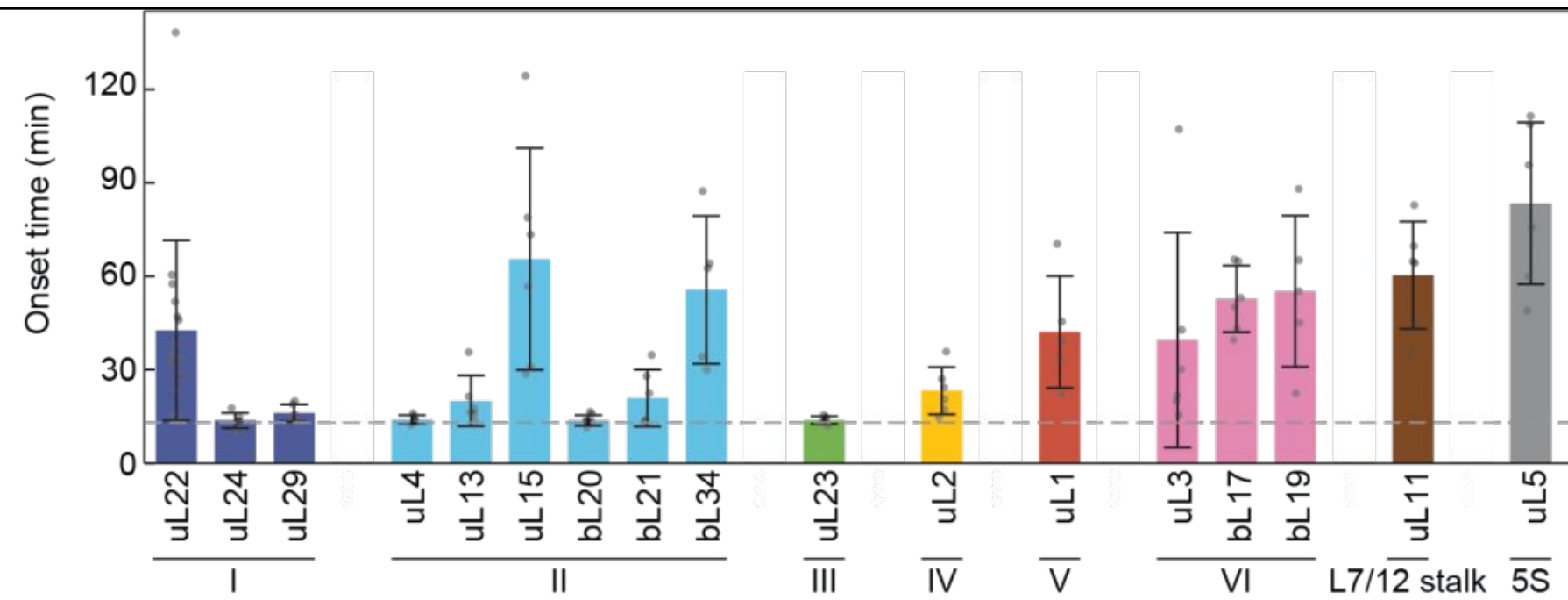

Figure S8: Onset time of coupled synthesis and assembly of all LSU components. The average onset time (Supporting Information) and standard deviation across at least 4 repeats measured for each r-protein-HA in the presence of all r-protein genes based on the raw data in Figure S7.

1

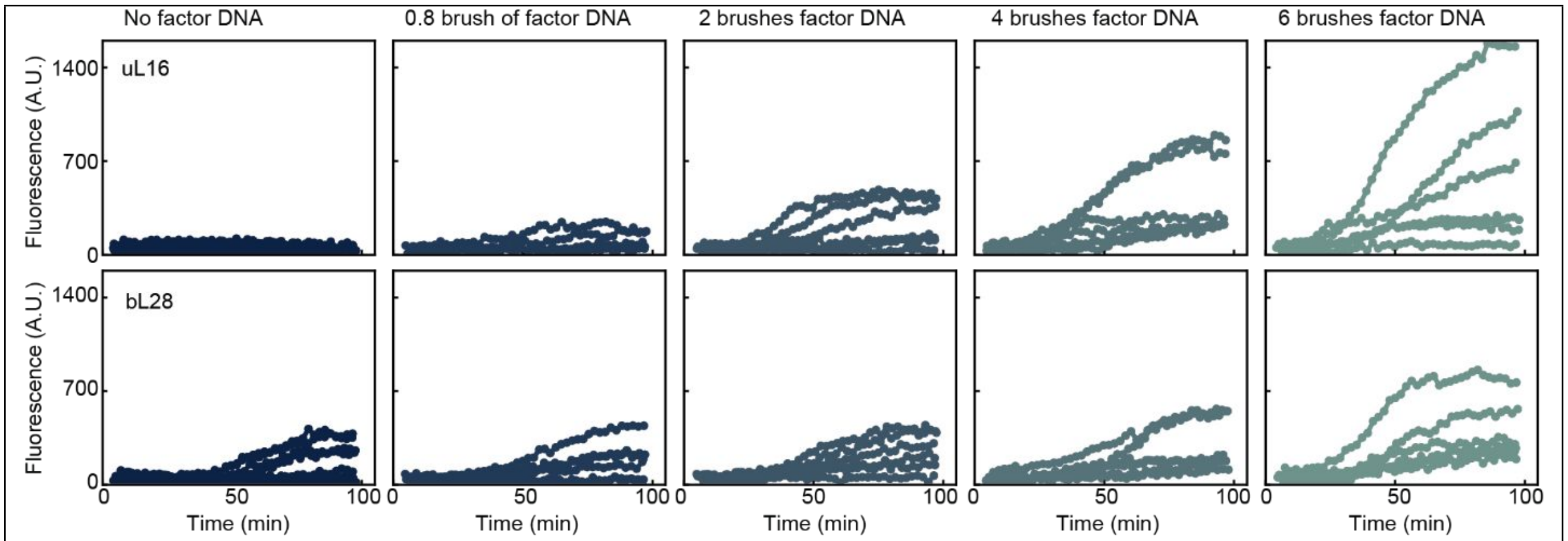

Figure S9: Titration of EngA and ObgE genes brushes. Raw dynamics of data in Figure 5D, E for uL16 and bL28 synthesis and assembly, with differing number of DNA brushes coding for EngA and ObgE. Each panel contains the dynamics of replicas of the same DNA brush setup.

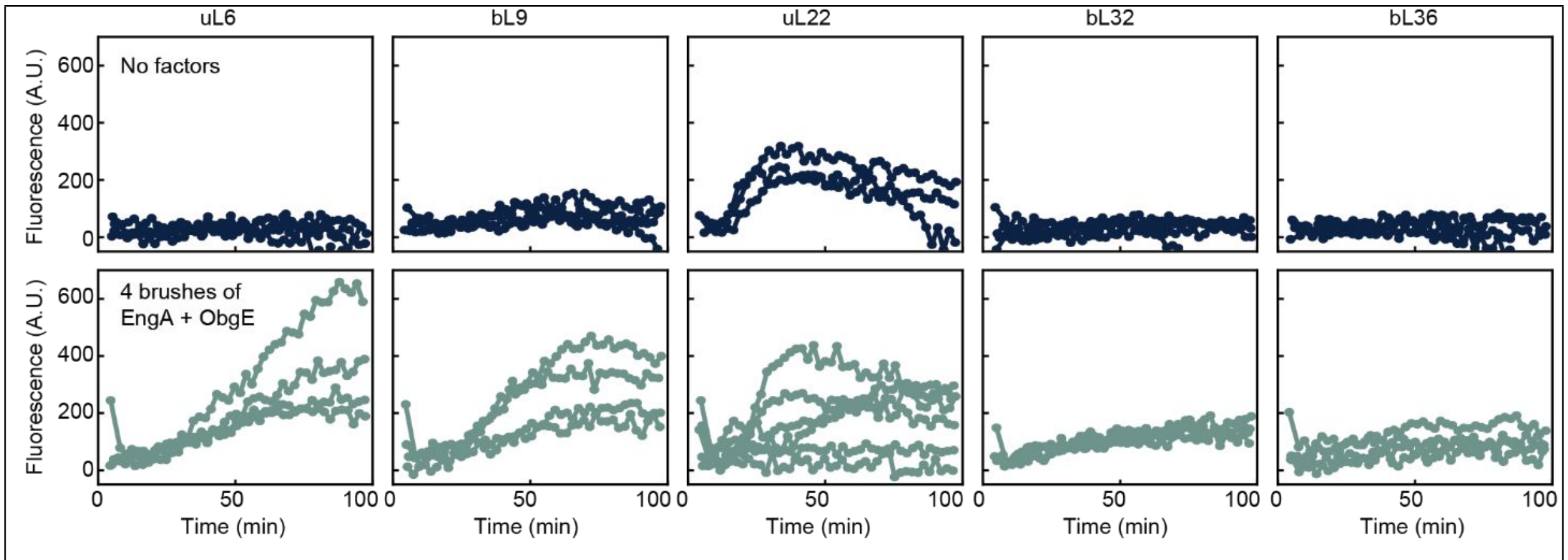

Figure S10: Effect of EngA and ObgE on synthesis and assembly of several late r-proteins. Raw dynamics of the data shown in Figure 6C. Each panel contains the dynamics of replicas of the same DNA brush setup.

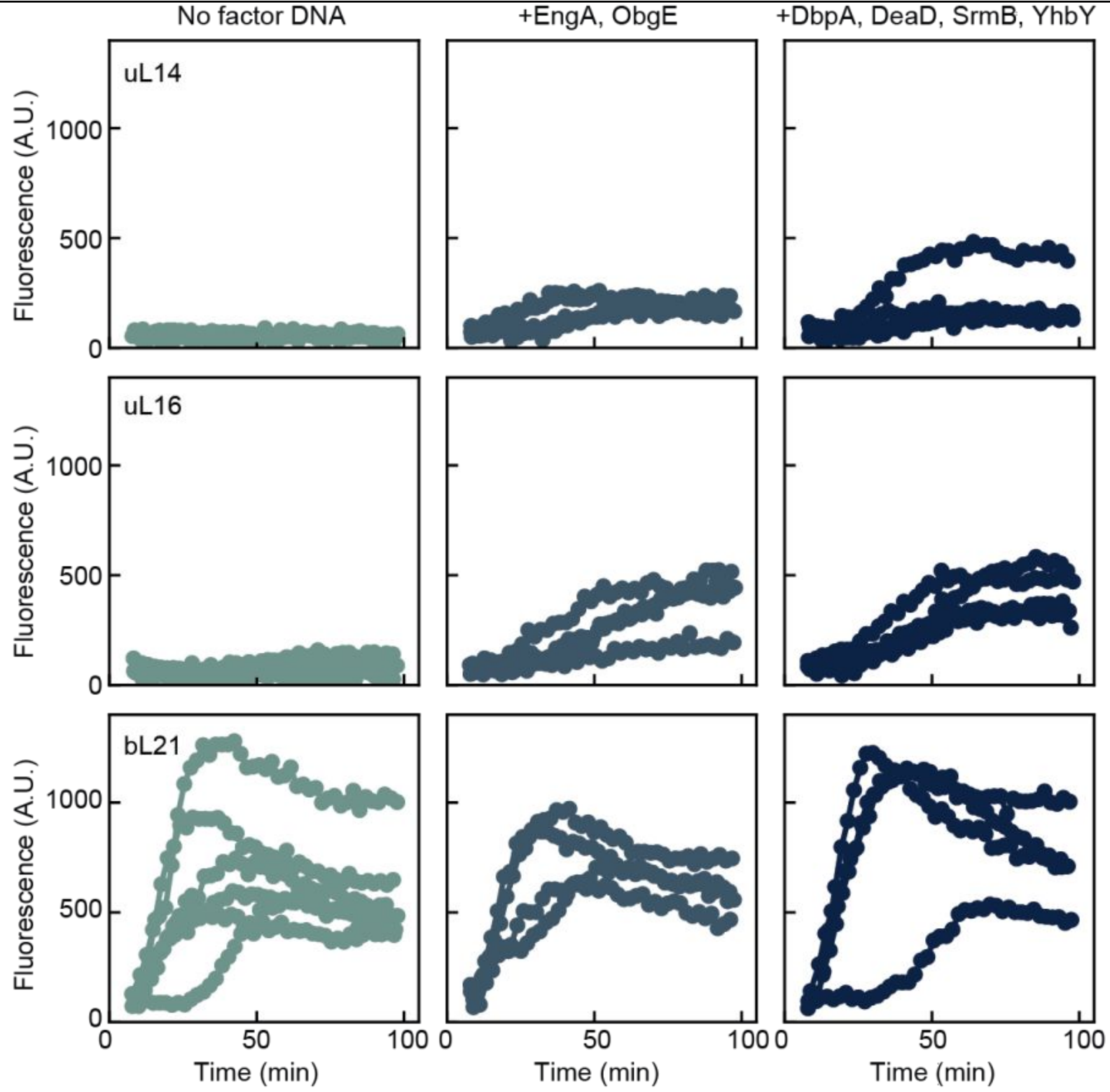

Figure S11: Synthesis and assembly of uL14, uL16 and bL21-HA to the 23S under assembly conditions, in the presence and absence of either genes coding for EngA and ObgE or for DbpA, DeaD, SrmB, YhbY. Raw dynamics of the data shown in Figure 6E. Each panel contains the dynamics of replicas of the same DNA brush setup.

References

1. Walker, S. C., Avis, J. M. & Conn, G. L. General plasmids for producing RNA in vitro transcripts with homogeneous ends. *Nucleic Acids Res.* **31**, e82 (2003).
2. Filonov, G. S., Moon, J. D., Svensen, N. & Jaffrey, S. R. Broccoli: Rapid Selection of an RNA Mimic of Green Fluorescent Protein by Fluorescence-Based Selection and Directed Evolution. *J. Am. Chem. Soc.* **136**, 16299–16308 (2014).
3. Buxboim, A. *et al.* A single-step photolithographic interface for cell-free gene expression and active biochips. *Small* **3**, 500–510 (2007).
4. Vonshak, O. *et al.* Programming multi-protein assembly by gene-brush patterns and two-dimensional compartment geometry. *Nat. Nanotechnol.* **15**, 783–791 (2020).
5. Filonov, G. S., Kam, C. W., Song, W. & Jaffrey, S. R. In-gel imaging of RNA processing using broccoli reveals optimal aptamer expression strategies. *Chem. Biol.* **22**, 649–60 (2015).
